# Interaction with the carboxy-terminal tip of SSB is critical for RecG function in *E. coli*

**DOI:** 10.1093/nar/gkad162

**Published:** 2023-03-13

**Authors:** Nina J Bonde, Camille Henry, Elizabeth A Wood, Michael M Cox, James L Keck

**Affiliations:** Department of Biomolecular Chemistry, University of Wisconsin-Madison, Madison, WI 53706, USA; Department of Biochemistry, University of Wisconsin-Madison, Madison, WI 53706, USA; Department of Biochemistry, University of Wisconsin-Madison, Madison, WI 53706, USA; Department of Biochemistry, University of Wisconsin-Madison, Madison, WI 53706, USA; Department of Biochemistry, University of Wisconsin-Madison, Madison, WI 53706, USA; Department of Biomolecular Chemistry, University of Wisconsin-Madison, Madison, WI 53706, USA

## Abstract

In *Escherichia coli*, the single-stranded DNA-binding protein (SSB) acts as a genome maintenance organizational hub by interacting with multiple DNA metabolism proteins. Many SSB-interacting proteins (SIPs) form complexes with SSB by docking onto its carboxy-terminal tip (SSB-Ct). An alternative interaction mode in which SIPs bind to PxxP motifs within an intrinsically-disordered linker (IDL) in SSB has been proposed for the RecG DNA helicase and other SIPs. Here, RecG binding to SSB and SSB peptides was measured *in vitro* and the RecG/SSB interface was identified. The results show that RecG binds directly and specifically to the SSB-Ct, and not the IDL, through an evolutionarily conserved binding site in the RecG helicase domain. Mutations that block RecG binding to SSB sensitize *E. coli* to DNA damaging agents and induce the SOS DNA-damage response, indicating formation of the RecG/SSB complex is important *in vivo*. The broader role of the SSB IDL is also investigated. *E. coli ssb* mutant strains encoding SSB IDL deletion variants lacking all PxxP motifs retain wildtype growth and DNA repair properties, demonstrating that the SSB PxxP motifs are not major contributors to SSB cellular functions.

## INTRODUCTION

Single-stranded (ss) DNA is a ubiquitous intermediate in nearly every genome maintenance pathway in dividing cells. In bacteria, ssDNA-binding proteins (SSBs) play two essential roles in mediating reactions involving ssDNA. First, SSBs bind ssDNA with high affinity, melting secondary structures to allow polymerases or other proteins access to the DNA while also protecting ssDNA from degradation. Second, SSBs interact with a large network of partner proteins, forming genome maintenance hubs at which multiple DNA replication, recombination, and repair factors assemble on genomic targets (1–3).

**Figure 1.**
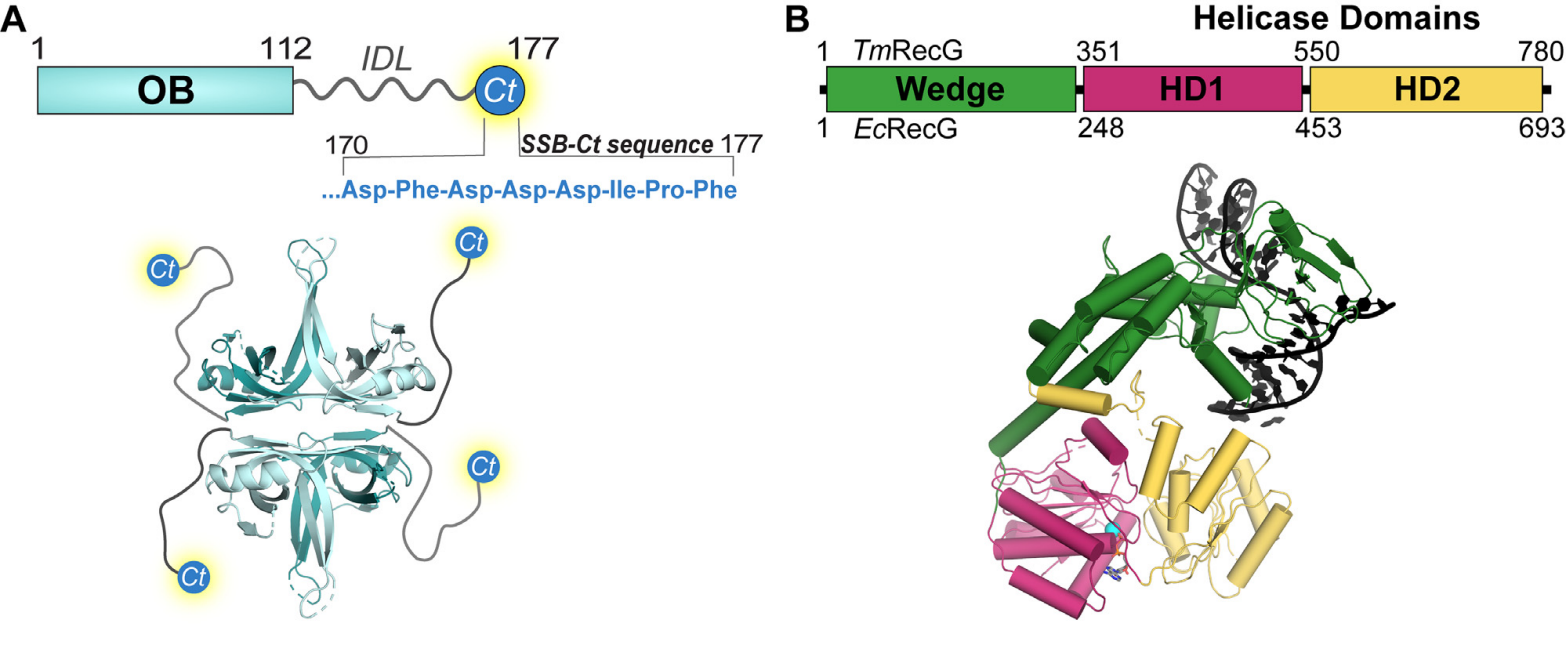
SSB and RecG domain layouts and structures. (**A**) *E. coli* SSB domain schematic and crystal structure with the unresolved IDL and SSB-Ct drawn in (PDB ID: 1SRU) (31). (**B**) *T. maritima* and *E. coli* RecG domain schematics and *T. maritima* RecG crystal structure (PDB ID: 1GM5) with branched DNA and Mg-ADP (shown as sphere-stick) (74). The wedge domain is represented in green and helicase domains (HD) 1 and 2 are represented in pink and yellow, respectively.

The prototypical SSB from *Escherichia coli* contains two structural elements: an N-terminal oligosaccharide/oligonucleotide binding (OB) domain and a C-terminal tail (Figure 1A). The OB domain mediates homotetramerization of the protein and DNA binding. Depending upon solution conditions, *E. coli* SSB binds between 35 and 65 ssDNA bases per tetramer and ssDNA binding can be highly cooperative, allowing SSB to coat ssDNA (4–7). *E. coli* SSB interacts with a network of ∼20 different DNA metabolism proteins via its C-terminal tail (8–30). The SSB tail is comprised of a poorly conserved intrinsically-disordered linker (IDL), which is unresolved in crystal structures (31–34), and a highly conserved, amphipathic C-terminal tip termed the SSB-Ct (-Asp-Phe-Asp-Asp-Asp-Ile-Pro-Phe) (Supplementary Figure S1).

Most SSB-interacting proteins (SIPs) studied to date appear to share a conserved mode of SSB recognition in which they dock onto the SSB-Ct. Several SIPs have been co-crystalized with SSB-Ct peptides and their SSB-binding pockets have been validated in biochemical and/or genetic studies (10,12,19,20,35). SSB-Ct binding pockets have also been successfully predicted through computational methods (36–39). Typical SSB-Ct binding sites contain three shared features: a hydrophobic pocket, where the side chain of the C-terminal Phe residue of SSB is buried; a basic lip, comprised of Arg, Lys and/or His residues that anchor the alpha-carboxyl group of the SSB C-terminus; and a basic ridge, where side chains from basic residues interact with Asp residues of the SSB-Ct. Deletion of the C-terminal-most Phe residue on SSB (SSBΔF) or the entire SSB-Ct motif (SSBΔC8) eliminates interactions between SSB and SIPs *in vitro* (8,9,11,12,14,16,18,20,22,27,29,30,40) and is lethal to *E. coli* (41,42). Strains carrying the *ssb-113* mutation, which substitutes a Ser for the penultimate Pro of the SSB-Ct, exhibit DNA damage hypersensitivity and temperature-sensitive growth (43–47). The SSB113 protein or peptide has a greatly reduced affinity for SIPs (8,10,12–14,18–20,29).

While roles for the SSB OB domain and SSB-Ct have been well-defined, investigations into potential roles of the IDL have been initiated more recently (41,48–51). Unlike the OB and SSB-Ct elements, the IDL is not well conserved across bacterial SSBs (Supplementary Figure S1) and its length varies greatly from species to species (41). *In vitro* studies have shown that the IDL is important for cooperative ssDNA binding (41) and for phase separation of SSB (50,52). However, plasmid complementation experiments have demonstrated that *E. coli* cell viability is maintained even when large segments of the IDL (47 or 51 of 57 residues) are removed from SSB (41,42). Similarly, *ssb* mutants in which fluorescent domains have been inserted into the IDL retain function *in vitro* and *in vivo* (53). These findings suggest that the SSB IDL is largely dispensable in *E. coli*. Nonetheless, direct roles for the IDL in SSB/SIP interactions have been proposed (48,49,51,54–57). These studies posit that PxxP motifs within the IDL bind to OB domains in SIPs, including RecG DNA helicase, and that the SSB-Ct plays an indirect role in SSB/SIP interactions. PxxP motifs mediate interactions with Src Homology 3 domains in some eukaryotic systems (58–61), which sets a precedent for possible PxxP motif protein interactions in bacteria. Evidence to support multiple modes of SSB recognition by SIPs would greatly impact our understanding of how SSB differentiates among its large network of partner proteins to successfully coordinate replication or repair process and recruit the appropriate genome maintenance enzymes to DNA.

RecG is a superfamily-2 DNA helicase and double-stranded DNA translocase that has been implicated in several DNA repair roles in bacteria (62–69). RecG binds and unwinds a variety of DNA substrates *in vitro* but exhibits a preference for junction DNA (70,71). *E. coli* strains lacking *recG* are sensitive to ultraviolet (UV) light and chemical DNA damaging agents (72) and are prone to filamentation and chromosomal partitioning defects (73). Loss of *recG* also leads to over-replication of the chromosome in the terminus region and at sites of double-strand DNA breaks (67,68). A crystal structure of RecG from *Thermotoga maritima* showed that the protein is comprised of an N-terminal ‘wedge’ domain involved in DNA junction binding/unwinding and two RecA-like helicase domains (HD1 and HD2) that function together as a molecular motor (Figure 1B) (74).

A physical interaction between RecG and SSB has been reported (29). While RecG/SSB complex formation requires an intact SSB-Ct, it has been proposed that PxxP motifs from the SSB IDL dock onto an OB fold within the RecG wedge domain and that the SSB-Ct plays an indirect role in the interaction (48,49). Evidence to support this model primarily comes from experiments in which RecG and SSB (or variants) simultaneously overexpressed in *E. coli* are co-purified using an affinity tag present on one of the two proteins. However, the interaction between RecG and SSB has not been characterized with purified proteins using methods that directly measure the stability of the complex or that identify interaction sites on RecG and SSB. SSB can modulate the activity of partner proteins and can recruit them to sites of action in cells, therefore understanding how RecG interacts with SSB could provide important insights into RecG function.

Our study uses biochemical and genetic approaches to answer several questions about the RecG/SSB complex: (i) Does purified *E. coli* RecG bind to the SSB-Ct, SSB IDL or both regions of SSB? (ii) Where is the SSB binding site on RecG? and (iii) Does destabilization of the RecG/SSB interaction affect RecG activity *in vivo*? Two different equilibrium binding assays using purified components show that RecG binds directly and specifically to the SSB-Ct with a dissociation constant of 3–4 μM. No binding to an IDL peptide is detected. Yeast two-hybrid analysis of a putative SSB-Ct binding site on the RecG HD2 domain predicted by a model of the complex shows that charge-reversal mutations of any of three residues in the predicted site (Arg474, Arg467 or Arg614) destabilize the RecG/SSB complex. The purified Arg474Glu RecG variant was unable to bind SSB *in vitro*, but it retained wildtype levels of DNA-dependent ATPase activity, consistent with a specific role for Arg474 in complex formation with SSB. In contrast, RecG wedge domain variants that had been proposed to bind the IDL PxxP motifs (48,49) retain interaction with SSB in two-hybrid and *in vitro* interaction assays. Mutation of the chromosomal *recG* gene in *E. coli* to encode RecG SSB-binding pocket variants sensitized the cells to UV light and led to constitutive SOS induction for a subset of the changes. The cumulative results show that RecG is a canonical SIP that interacts directly with the SSB-Ct using a site on the HD2 domain to form a complex that is important for RecG function *in vivo*.

As an extension of this study, we examined the effects of deleting large regions of the SSB IDL from the native *ssb* gene. Deleting residues 151–166, 139–164 or 130–166 of the IDL, which remove either two or all three IDL PxxP motifs in *E. coli* SSB, has no measurable effect on cell growth or sensitivity to DNA damaging agents in the mutants. Cells expressing an SSB IDL deletion variant lacking residues 120–166 have a slight fitness defect, which can be reversed when 10 IDL residues are re-introduced in randomized order. These results suggest that *E. coli* SSB requires a minimal IDL length, but not a specific sequence, and that the IDL functions as a spacer between the ssDNA-binding OB domain and the protein-binding SSB-Ct motif *in vivo*.

## MATERIALS AND METHODS

### Strain construction

All bacterial strains used in this study are *E. coli* K-12 MG1655 or derivatives (Supplementary Table S1). Primers used for strain construction are listed in Supplementary Table S2. EAW1515, EAW1516, EAW1517 and EAW1518 chromosomally encode *ssb* IDL mutations *ssbΔ139–164*, *ssbΔ151–166*, *ssbΔ130–166*, *ssbΔ120–166*, respectively, which were designed and described previously (41). The strains were generated by PCR amplifying EAW1169 (53) with a primer downstream of the FRT-Kan-FRT site and a primer with 50 bases homologous to the region upstream of the site of deletion and 18–20 bases downstream of the site of deletion. PCR products were gel purified and integrated onto the chromosome using λRED recombination (75). NJB48 was generated similarly except that EAW1518 was used as the PCR template strain and amplified with a primer containing the bases to be inserted. All mutant constructs remain under the control of the native *ssb* promoter. Strain genotypes were confirmed by PCR, Sanger sequencing, and whole genome sequencing.

Additional strains used in growth competition assays were generated by P1 transduction (76), where EAW214 (Δ*araBAD*) was transduced with P1 phage grown on EAW1515, EAW1516, EAW1517, EAW1518 or NJB48. Strains were confirmed by PCR and Sanger sequencing.

EAW1650, EAW1651, EAW1693 and EAW1707 chromosomally express *recG* binding pocket mutants *recG-R474E*, *recG-R467E*, *recG-R614E* and *recG-R484E* under the control of the native *recG* promoter, respectively. Plasmids encoding the desired *recG* mutant were used as the template in a PCR with recGus and recGdsBam primers. Another PCR was run with pEAW507 (carrying a mutant FRT-Kan^R^-wildtype FRT cassette) as template and recGend and recGafter primers. The two PCR products have 42 bp overlapping regions and were digested with DpnI and gel purified. The gel purified PCR products were used as the template in a second PCR with recGus and recGafter primers. The resulting PCR product was gel purified and electroporated into EAW505/pKD46. Strains were confirmed by PCR and Sanger sequencing.

### Protein purification

RecG was expressed in STL2669/pT7pol26 (a gift from Dr Susan Lovett, Brandeis University) with overexpression plasmid pEAW111 (77). Eight liters of Luria Broth (LB) culture supplemented with 100 μg/ml ampicillin and 40 μg/ml kanamycin were induced at an OD_600 nm_ of 0.4 by the addition of IPTG to a final concentration of 0.4 mM and incubated for 3 h at 37°C. Cells were pelleted and flash frozen for storage at −80°C. The cell pellet was resuspended in 25% sucrose solution and lysed by lysozyme (0.2 mg/ml) at room temperature for 15 min, followed by sonication. Ammonium sulfate was added to 25% saturation to the clarified lysate and stirred at 4°C for 3 h. The mixture was pelleted, and ammonium sulfate was added to bring the supernatant to 40% saturation and stirred overnight at 4°C. The mixture was pelleted, and the pellet was resuspended in Buffer A (20 mM Tris−HCl, pH 7.6, 0.1 mM EDTA, 10% glycerol, 1 mM DTT, 50 mM KCl) and loaded onto a DEAE column. Fractions containing RecG were loaded onto a Source 15Q column. Fractions containing RecG were loaded onto a Source 15S column and eluted with a gradient from 50 mM KCl to 1 M KCl. Fractions containing RecG were tested for nucleases, and nuclease free fractions were pooled and dialyzed into storage buffer (20 mM Tris−HCl, pH 7.6, 0.1 mM EDTA, 20% glycerol, 2 mM DTT, 500 mM KCl) or dialyzed into buffer for isothermal titration calorimetry (ITC) experiments. Purified RecG appeared as a single band on SDS-PAGE gels and was quantified using monomer extinction coefficient ε_280 nm_ = 5.022 × 10^4^ M^−1^ cm^−1^.

*In vivo* assembly mutagenesis (78) was used to introduce the R474E or R95A mutation into RecG overexpression plasmid pEAW111 to create variant overexpression plasmids pNJB3 and pEAW1324, respectively. RecG R474E or RecG R95A were expressed in STL2669/pT7pol26 and purified using the same purification scheme as wildtype RecG. The purified proteins appeared as single bands on SDS-PAGE gels and were quantified using monomer extinction coefficient ε_280 nm_ = 5.022 × 10^4^ M^−1^ cm^−1^. The RecG R95A preparation included a very weak nickase contaminant.

SSB and SSBΔF were purified as previously described (79) and quantified using monomer extinction coefficient ε_280 nm_ = 2.8 × 10^4^ M^−1^ cm^−1^. SSBΔ120–166 was a kind gift from Dr Alexander Kozlov and Dr Timothy Lohman.

### Fluorescence anisotropy

**Figure 2.**
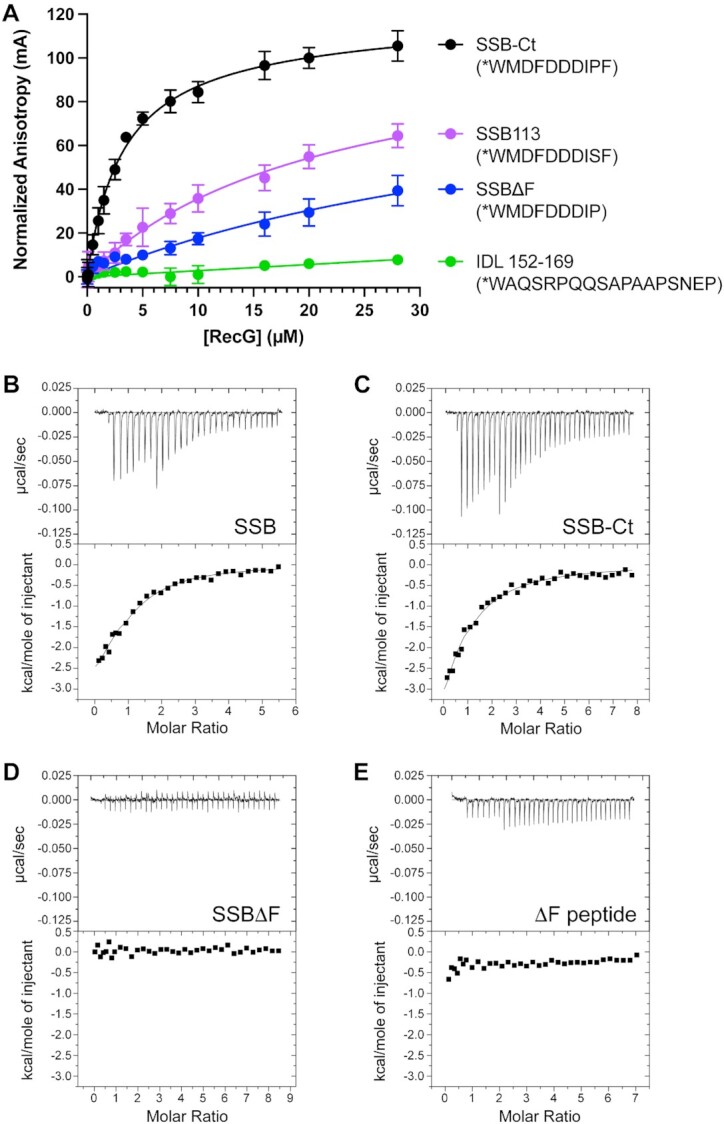
RecG binds directly and specifically to the SSB-Ct. (**A**) Fluorescence anisotropy binding isotherms for RecG binding to SSB-Ct (black), SSB113 (violet), SSBΔF (blue), or SSB IDL (green) peptides. Peptide sequences are listed in parentheses with * representing an N-terminal 6-FAM label. Anisotropy values are normalized to the reading at 0 μM RecG. The mean value of experiments performed in triplicate is graphed with error bars representing one standard deviation from the mean. (B–E) ITC analysis of RecG/SSB binding. Thermograms (top) and binding isotherms (bottom) of titrations of (**B**) full-length SSB, (**C**) SSB-Ct peptide, (**D**) SSBΔF or (**E**) SSBΔF peptide into RecG solutions.

SSB peptides (sequences listed in Figure 2) were designed with an N-terminal tryptophan residue for quantification and N-terminal 6-FAM label (Genscript). RecG was incubated with 0.2 M potassium glutamate, 1 mM DTT, 0.1 μg/ml BSA, 1 × RecA buffer (25 mM Tris-acetate, pH 7.6, 5% w/v glycerol, 3 mM potassium glutamate, 10 mM magnesium acetate, 1 mM DTT), and 10 nM labeled peptide at room temperature for 30 min. Fluorescence polarization (FP) values were measured at 25°C using a Beacon 2000 Fluorescence Polarization System. Polarization values were normalized to the FP value measured at 0 nM RecG. Normalized FP was converted to normalized anisotropy. Experiments were performed in triplicate, and the data were fit to a one site binding model ($Y\ = {B_{max}}\ \times X \div ( {{K_D} + X} )$, where *X* is ligand concentration, *Y* is normalized anisotropy, *B*_max_ is the maximum anisotropy, and *K*_D_ is the dissociation constant) using GraphPad Prism Software. Graphs plot the average normalized anisotropy value from triplicate experiments with error bars representing one standard deviation from the mean.

### Isothermal titration calorimetry

Isothermal titration calorimetry (ITC) experiments were performed with a MicroCal/Malvern VP-ITC. SSB and RecG were dialyzed into the same buffer (20 mM Tris−HCl, pH 8.0, 150 mM NaCl, 10% glycerol, 1 mM EDTA, 1 mM β-mercaptoethanol). SSB peptides (unlabeled, Genscript) were dissolved in the buffer used for dialysis. ITC experiments were performed at 25°C with stirring (307 rpm). Injections were varied in volume: 1 × 1 μl, 7 × 4 μl, and 22 to 27 × 8 μl. Full-length SSB proteins or peptides were injected into wildtype RecG (7.41 μM), RecG R474E (12.9 μM), or RecG R95A (6.67 μM). All protein concentrations are expressed as concentration of monomers. To account for the heat of dilution, wildtype SSB (263 μM), SSBΔF (335 μM), SSB-Ct peptide (WMDFDDDIPF, 308 μM), and ΔF peptide (WMDFDDDIP, 278 μM) were injected into buffer (Supplementary Figure S2) and subtracted from titrations with RecG. ITC data were analyzed using Origin software (MicroCal), where data were fit to a one-site binding model to determine the binding stoichiometry, dissociation constant, Δ*H*°, Δ*G*° and *T*Δ*S*° for complex formation.

### AlphaFold prediction and conservation analysis

AlphaFold2 (80) prediction of a 1:1 RecG:SSB complex was completed using AlphaFold-Multimer (81). Full-length *E. coli* SSB and RecG protein sequences were used as the input, and the prediction was run using the reduced database data preset. Images show the predicted complex structure after Amber relaxation. Resulting models were imported into PyMOL to generate figures.

Conservation analysis of 300 of the closest sequences to *E. coli* RecG was completed using ConSurf (82,83), with the protein sequence used as the input. The analysis was run using the default settings (homolog search algorithm: HMMER with 1 iteration and 0.0001 E-value cutoff, protein database: UNIREF-90, Maximal %ID: 95, Minimal %ID: 35%), and conservation scores were visualized on the RecG AlphaFold2 model using PyMOL.

### Yeast two-hybrid assay

SSB, RecG or RecG variants were N-terminally fused to the DNA binding domain (pGBD) or activation domain (pGAD) of the Gal4 transcription factor. Resulting plasmids (Supplementary Table S3) were transformed (84) in pairs into *Saccharomyces cerevisiae* strain pJ69-4a (85) and selected for on synthetic dextrose (SD) Leu- and Trp-deficient plates. Transformants were inoculated into SD Leu- and Trp-deficient liquid medium and grown at 30°C overnight rotating. Saturated overnight cultures were diluted to an OD_600 nm_ of 1.0 in 2% glucose and serially diluted 10-fold in 2% glucose. Dilutions were spotted (10 μl) onto SD His- and Ade-deficient plates and grown at 30°C for 4–5 days. Plates were imaged on a Fotodyne gel doc. Site-directed mutagenesis of RecG in pGBD was performed by *in vivo* assembly mutagenesis (78) of pEAW1156.

### ATP hydrolysis assay

ATPase rates for wildtype and variant RecG proteins were determined using a coupled spectrophotometric assay (86). ATP hydrolysis reactions were conducted with 150 nM RecG in 1 × RecA buffer, supplemented with 1 mM DTT, 2.2 mM phosphor(enol)pyruvate, 10 units/ml pyruvate kinase, 10 units/ml lactate dehydrogenase, 2 mM NADH, and 3 mM ATP with varying concentrations of ΦX174 RF I (New England Biolabs) DNA at 37°C. DNA concentrations are represented in μM nucleotides (nt). *A*_380 nm_ measured the conversion of NADH into NAD^+^. Measurements were taken every 20–40 s on a Cary UV-Vis spectrophotometer. ATP hydrolysis rates were calculated by dividing the slope of A_380 nm_ measurements over time over the linear region by ε_380 nm_ = 1.21 mM^−1^ cm^−1^ for NADH and the cell path length (1 cm).

### Growth curves

Overnight cultures of strains of interest were diluted 1:100 in LB and grown at 37°C with shaking at 200 rpm to an OD_600 nm_ of 0.1. Cultures were diluted 1:1 in LB in 96-well plates, and the OD_600 nm_ of each well was measured every 10 min over a 24-h incubation at 37°C on a microplate reader (BioTek Synergy H1). Growth curves plot the average OD_600 nm_ of biological triplicates over time with error bars representing one standard deviation from the mean.

### Western blots

SSB western blots were carried out and quantified as previously described (53). Briefly, cultures of MG1655 or *ssb* IDL mutant strains were grown at 37°C with shaking to an OD_600 nm_ of 0.2. One ml of each culture was pelleted and resuspended in 1 × cracking buffer (CB) (0.5 M Tris–HCl, pH 6.8, 5% SDS, 1% glycerol, 0.5% β-mercaptoethanol, 0.005% bromophenol blue) so that the volume of CB used for pellet resuspension resulted in 100 μl of each resuspension containing the equivalent of 1 ml of cells at OD_600 nm_ = 1.0. Purified *E. coli* SSB and SSBΔ120–166 were also diluted in 1 × CB to 0.1, 0.05, or 0.025 μM final concentrations for western blot quantification standards. Samples were denatured for 10 min at 95°C, and 10 μl of each sample (undiluted or diluted 3-fold) were loaded onto a 15% SDS-PAGE gel. Proteins were transferred to a nitrocellulose membrane for 1 h at 50 V. Membranes were blocked in 5% milk, 1 × phosphate buffered saline (PBS) (137 mM NaCl, 2.7 mM KCl, 10 mM Na_2_HPO_4_, 1.8 mM KH_2_PO_4_, 1 mM CaCl_2_, 0.5 mM MgCl_2_), 0.05% Tween (5% Milk PBS-T) for 30 min at room temperature and incubated in 5% Milk PBS-T with a 1:600 dilution of the primary antibody (polyclonal anti-SSB from rabbit) for 1.5 h. Membranes were washed in PBS-T and incubated with 1:3000 secondary antibody (anti-rabbit-HRP goat from Abcam) in 1 × PBS-T for 45 min. Membranes were washed in 1 × PBS and visualized using SuperSignal West Femto PLUS (Thermo Scientific). Blots were imaged on an iBright Imaging System. Quantifications were performed as previously described (53) in biological triplicate.

Polyclonal antibodies against *E. coli* RecG were raised in rabbit (Labcorp). RecG western blots were preformed similarly to SSB western blots with the following modifications. Cultures of MG1655 or strains expressing *recG* mutants were pelleted and resuspended in 1 × CB so that the volume of CB used for pellet resuspension resulted in 100 μl of each resuspension containing the equivalent of 1 ml of cells at OD_600 nm_ = 2.0. Ten μl of each cell sample were loaded onto a 4–15% Mini-PROTEAN TGX gel (Bio-Rad). Proteins were transferred to a nitrocellulose membrane for 90 mins at 55 V. After blocking, the membrane was incubated with a 1:200 dilution of primary antibody at 4°C overnight.

### DNA damage sensitivity spot plate assays

MG1655, *recG*, and *ssb* IDL mutant strains were tested for sensitivity to UV light, ciprofloxacin, or mitomycin C. A Δ*recA* strain (EAW20) was used as a sensitive control in the *ssb* IDL experiments. Overnight cultures set in biological triplicate were used to inoculate fresh LB. Cells were grown at 37°C with shaking to an OD_600 nm_ of 0.2 and serially diluted 10-fold in 1 × PBS. Ten μl of each dilution was spotted onto square LB agar plates (for UV) or LB plates containing DNA damaging agents (ciprofloxacin or mitomycin C) poured fresh and kept in the dark. UV sensitivity plates were exposed to shortwave light (254 nm) using a Spectrolinker XL-1000 UV crosslinker (Spectronics Corp) and incubated at 37°C overnight in the dark. Plates were imaged on an iBright Imaging System. UV sensitivity was quantified as previously described (87). Briefly, dilutions of each strain were plated onto an LB plate exposed to 0, 50 or 100 J/m^2^. Plates were incubated under foil at 37°C overnight, and colonies were counted to obtain colony forming units (CFU)/ml and percent relative survival values.

### SOS induction assay

To measure the induction of SOS, MG1655, EAW505 (Δ*recG*), *recG* mutant strains and *ssb* IDL mutant strains of interest were transformed separately with pQBI63 or pEAW903. Plasmid pEAW903 contains SuperGlo GFP under the control of the *recN* promoter. Overnight cultures of transformants were grown in biological triplicate. Cultures were diluted 1:100 in LB supplemented with 100 μg/ml ampicillin and grown to an OD_600 nm_ of 0.1. Cultures were diluted 1:1 in LB with ampicillin in a 96-well plate. The OD_600 nm_ and fluorescence at 515 nm (excitation at 488 nm) were measured for each well every 10 min for 24 h in a H1 Synergy Biotek plate reader. Transformed *recG* mutant strains were also imaged under a microscope as described below after 5 h of growth to quantify cell length and SOS induction.

### Growth competition (fitness) assay

Strain fitness was evaluated using a modified growth competition assay (87,88). Overnight cultures of Δ*araBAD* and *araBAD*^+^ strains were set in biological triplicate. To start the assay, equal volumes of saturated Δ*araBAD* and *araBAD*^+^ overnight cultures were mixed. LB was inoculated 1:100 with the mixed culture, followed by growth at 37°C with shaking. Cultures were reinoculated into fresh medium every 24 h. At 0, 24, 48 and 72 h of incubation, mixed cultures were serially diluted in 1 × PBS and plated on tetrazolium arabinose plates. Plates were incubated at 37°C overnight before counting white (*araBAD*^+^) and red (Δ*araBAD*) colonies. Growth competition graphs represent data collected in biological triplicate for each combination of *araBAD*^+^ and Δ*araBAD* strains with the average percentage of red and white colonies reported. One standard deviation from the mean is represented as error bars.

### Microscopy and image analysis

Saturated overnight cultures of strains of interest were diluted 1:100 in LB and grown at 37°C for 5 h. Cells were pelleted and resuspended in 1 × PBS to an OD_600 nm_ of ∼1.0 on ice. The cell membrane was stained by addition of FM4-64 to a final concentration of 6.6 mM approximately 15 min before imaging. Cells (2.5 μl) were sandwiched between a coverslip and a 1.5% agarose pad. Images were acquired on a Nikon Ti-eclipse N-STORM microscope and ORCA Flash 4.0 digital CMOS C13440 camera (Hamamatsu) at room temperature with 100x magnification using NIS-Elements software. Brightfield (6.5 V, 100 ms exposure) and fluorescence channels were imaged with lasers (561 nm equipped with Chroma Sedat Quad filter ET 605/52m and 488 nm equipped with Chroma Sedat Quad filter ET 525/36 ms) in epifluorescence configuration. Images were acquired in the 561 channel (6.5 V, 400 ms exposure). To measure SOS induction in strains transformed with pPrecN-sfgfp (pEAW903), images were also acquired in the 488 channel (6.5 V, 100 ms exposure). Imaging was completed in biological triplicate for each strain on separate days.

Images were analyzed using Fiji software (ImageJ) and the MicrobeJ plugin (89), where processing was done uniformly across the entire image. Raw images for each strain (4–5 per replicate) were concatenated, and the brightfield, FM, and GFP image stacks were auto-scaled. Hyperstacks were generated using the brightfield and GFP channel images. The brightfield stacks were inverted and all processed the same way to adjust the image contrast using the script (Brightfield MJ contrast.ijm), and manually re-adjusted by enhancing the contrast and dividing the signal over the entire field of view. Unprocessed images of the 488 channel were used to quantify GFP signal. FM 4–64 adjusted images or hyperstacks combining the brightfield adjusted images and GFP channels generated in MicrobeJ were used for the analysis. Cell outlines were detected in the brightfield using the setuprecg2.xml file. Cell outlines were manually sorted to remove cells with poor outline fitting or that were out of focus. Cell ID, length, and fluorescence intensity were exported from MicrobeJ as .csv files and plotted with GraphPad Prism software, where statistical analyses were generated. Dunn's test for multiple comparisons was used to evaluate statistical significance of cell length and mean fluorescence data. A minimum of 2000 cells were analyzed for each strain.

## RESULTS

### RecG binds directly and specifically to the SSB-Ct

Two models explaining how RecG interacts with SSB have been proposed - RecG could interact with the SSB-Ct (29) or RecG could interact with the IDL (48,49). To begin testing each model, we used an equilibrium fluorescence anisotropy assay to measure RecG binding to fluorescently labeled SSB-Ct or IDL peptides (Figure 2). Titration of RecG with the SSB-Ct peptide led to an increase in fluorescence anisotropy, which occurs when protein binding slows the tumbling rate of a labeled peptide (90). The data fit well to a one-site binding model with a dissociation constant (*K*_D_) of 3.1 ± 0.4 μM (Table 1). *K*_D_ values in the low micromolar range are typical for SSB/SIP complexes (10,12–14,19,23).

**Table 1.** FP and ITC derived binding parameters

		FP	ITC				
RecG	SSB	*K*_D_ (μM)	Stoichiometry	*K*_D_ (μM)	ΔH° (kcal/mol)	TΔS° (kcal/mol)	ΔG° (kcal/mol)
WT	SSB-Ct peptide	3.1 ± 0.4	0.92 ± 0.08	3.9 ± 0.8	−4.0 ± 0.5	3.4 ± 0.6	−7.4 ± 0.5
WT	SSBΔF peptide	>30	ND	ND	ND	ND	ND
WT	SSB113 peptide	23.0 ± 3.1	-	-	-	-	-
WT	IDL 152–169 peptide	ND	-	-	-	-	-
WT	WT	-	1.05 ± 0.07	2.6 ± 0.4	−3.0 ± 0.3	4.9 ± 0.5	−7.9 ± 0.3
WT	SSBΔF	-	ND	ND	ND	ND	ND
R474E	SSB-Ct peptide	>30	ND	ND	ND	ND	ND
R474E	WT	-	ND	ND	ND	ND	ND
R95A	SSB-Ct peptide	1.8 ± 0.4	-	-	-	-	-
R95A	WT	-	1.0*	1.7 ± 0.7	−2.1 ± 0.3	5.8 ± 0.4	−7.9 ± 0.2

ND = binding not detected. Parameters that were not measured are marked with a hyphen. *Denotes fixed parameter during curve fitting.

To test the sequence specificity of RecG binding to the SSB-Ct, we examined binding to two SSB-Ct peptide variants. Titrations of RecG with an SSB-Ct peptide lacking the C-terminal Phe residue (SSBΔF) revealed greatly weakened binding by RecG (*K*_D_ > 30 μM). Likewise, changing the Pro residue in the SSB-Ct motif to a Ser (SSB113) also weakened the complex (*K*_D_ = 23.0 ± 3.1 μM) (Figure 2A, Table 1). Similar specificity has been observed with other SIPs (8,10–14,18–20,43). Thus, RecG binds a fluorescently labeled SSB-Ct peptide with a stability and sequence specificity that parallels that observed for other SIPs.

In contrast to the results observed with the SSB-Ct, titration of RecG with the fluorescently labeled IDL peptide showed no binding, even at the highest concentration tested (Figure 2A, Table 1). The IDL peptide that was used included residues 152–169 of SSB, which has two of the three PxxP motifs in *E. coli* SSB. This peptide includes a PxxP motif that has been reported to be necessary for RecG binding (49) and it is identical to a peptide that was previously reported to bind to the AlkB protein (51). These data are inconsistent with the SSB IDL directly binding to RecG.

We next used ITC to measure the stoichiometry and thermodynamic properties of the RecG/SSB interaction. In this experiment, the heat evolved when full-length SSB or the SSB-Ct peptide binds RecG was measured and the data were fit to determine the binding stoichiometry, *K*_D_, Δ*H*°, Δ*G*° and *T*Δ*S*° for complex formation. Titrations of SSB or the SSB-Ct peptide with RecG produced remarkably similar binding isotherms and thermodynamic profiles (Figure 2B and C, Table 1). The data from both titrations fit well to one-site models with stoichiometries very close to 1.0 (0.92 ± 0.08 for SSB-Ct peptide, 1.05 ± 0.07 for each protomer in full-length SSB). Remarkably, the Δ*G*° for RecG binding to full-length SSB (−7.9 ± 0.3 kcal/mol) was within the margin of error for RecG binding to the SSB-Ct peptide (−7.4 ± 0.5 kcal/mol). Δ*H*° and *T*Δ*S*° values were also nearly indistinguishable for SSB and SSB-Ct binding, strongly suggesting that the SSB-Ct comprises the complete binding site for RecG and that other elements within SSB are not involved in complex formation. To examine the specificity of the interaction, RecG binding to the SSBΔF protein variant or the SSBΔF peptide, both of which lack the C-terminal-most Phe residue, was measured. No binding was observed in either case (Figure 2D and E). These results confirm an interaction between RecG and the SSB-Ct motif of SSB and indicate that RecG binding to the SSB-Ct is specific, necessary, and sufficient for RecG/SSB interaction.

### Identification of the SSB-binding pocket on the HD2 domain of RecG

**Figure 3.**
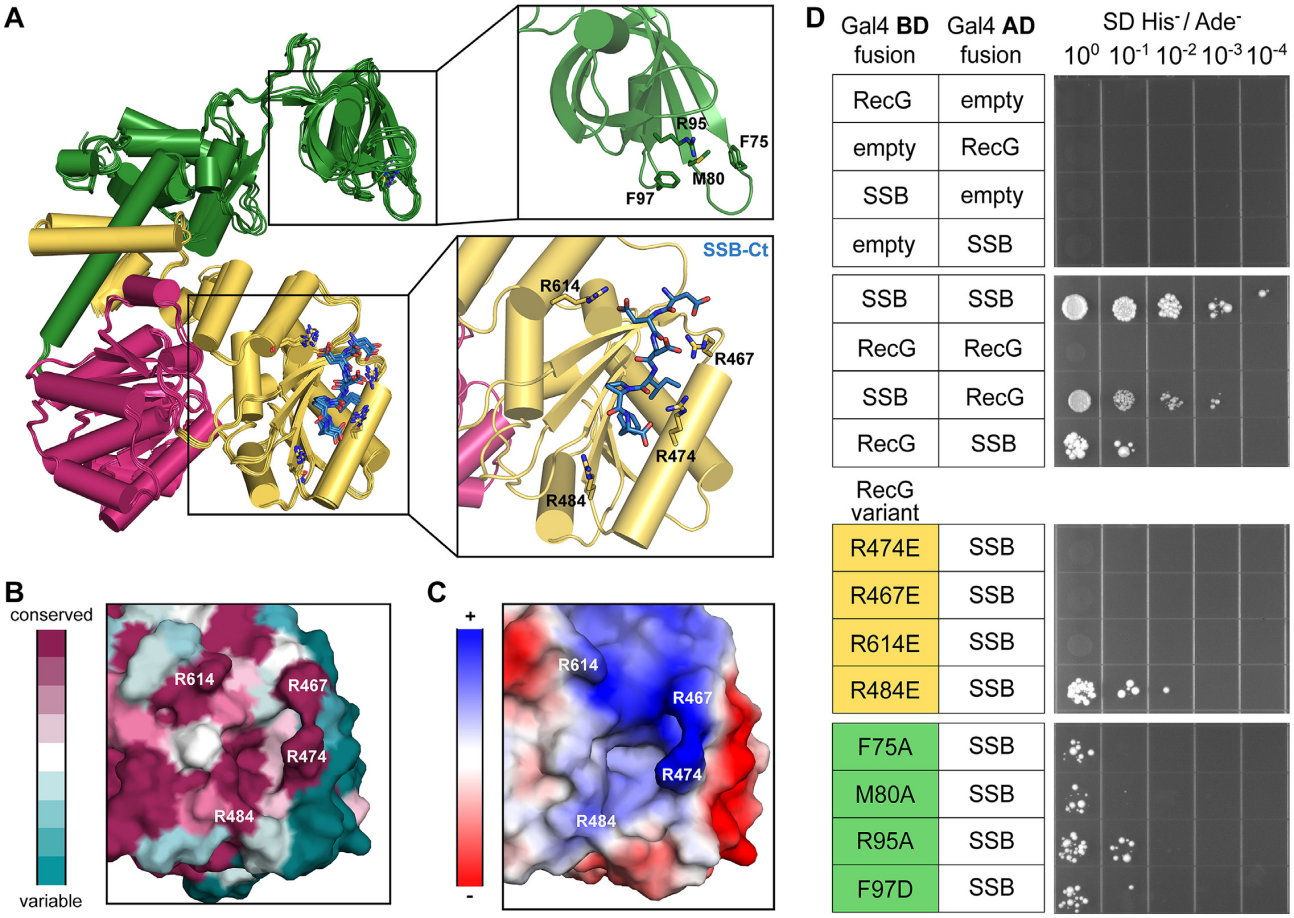
Prediction and identification of the SSB binding pocket on RecG. (**A**) Overlay of the five highest-confidence AlphaFold models of the RecG/SSB complex prediction. RecG is colored as in Figure 1 and the SSB-Ct is colored blue and shown as sticks (left). Zoomed-in views of the predicted wedge domain residues involved in SSB IDL binding (top right) and the predicted SSB-Ct binding pocket on one AlphaFold model (bottom right). RecG residues of interest are labeled and represented as sticks. (**B**) Conservation of residues in candidate binding pocket on RecG. (**C**) Electrostatic map of candidate binding pocket on RecG. (**D**) Yeast two-hybrid screen for interaction between SSB and RecG, RecG charge-reversal variants of conserved Arg residues in the candidate binding pocket, or RecG wedge domain variants.

To identify potential SSB binding sites on RecG, AlphaFold-Multimer complex prediction (80,81) was used to create models of the RecG/SSB complex. In each of the five highest-confidence models, the SSB-Ct motif of SSB docked against the same surface-exposed pocket in the HD2 domain of RecG (Figure 3A) whereas the SSB OB domain and IDL are in different positions in each model. Inspection of the putative SSB-Ct docking site revealed three Arg residues that appear well-positioned to interact with the alpha-carboxyl group of the terminal SSB-Ct Phe residue (R474) or the SSB-Ct Asp side chains (R467 and R614). This collection of residues is highly conserved among RecG proteins, and they combine to form an electrostatic structure that shares similarity with SSB-binding pockets observed in prior crystal structures of SIP/SSB-Ct complexes (Figure 3B and C) (10,12,19,20,35). A fourth Arg residue (R484) was also near the SSB-Ct docking site in the model but was not predicted to be directly involved in binding SSB (Figure 3A); this residue is used as a control in binding studies below. The predicted SSB binding site in the models does not overlap with the DNA binding surface of RecG (74) and is distinct from the proposed PxxP-binding site in the RecG wedge domain, which includes residues F75, M80, R95 and F97 (49).

To test the validity of the predicted SSB-binding site in the RecG HD2 domain, Arg-to-Glu charge reversal variants of RecG residues 474, 467 and 614 were created. A fourth charge-reversal variant of R484 was also tested as a control to determine whether an electrostatic change that is near, but not in, the putative SSB binding site would alter RecG binding to SSB. A yeast two-hybrid assay was used as an initial method to screen several RecG variants for interaction with SSB. In the two-hybrid assay, yeast were transformed with plasmids encoding SSB and RecG fused to the Gal4 activation and binding domains, respectively, and the RecG/SSB interaction was assessed by selecting for growth on selective medium plates. Cells in which RecG and SSB interact should grow whereas cells in which the interaction is disrupted by mutation should not. As anticipated, a strain transformed with plasmids expressing wildtype RecG and SSB Gal4 hybrids grow on selective medium whereas strains transformed with empty vector control plasmids do not (Figure 3D). Control strains transformed with plasmids that swap the activation and binding domains on RecG and SSB or where SSB is fused to both the activation and binding domains also confer growth, indicating that the specific arrangement of Gal4 domains with the bait and prey proteins does not alter the experiment. When plasmids encoding RecG R474E, R467E or R614E variants were substituted for RecG in the assay, the strains failed to grow, indicating that the mutations disrupted RecG/SSB interaction (Figure 3D). In contrast, growth was maintained in a strain expressing RecG R484E and SSB, indicating that the RecG/SSB interaction can tolerate an electrostatic change in the RecG HD2 surface that is not targeted to a residue predicted to directly bind SSB. These results are consistent with R474, R467 and R614 having roles in binding SSB as predicted by the AlphaFold models.

Notably, these results are inconsistent with a role for the RecG wedge domain in binding to SSB PxxP motifs. Such an interaction mode would have resulted in positive interactions for all of the RecG/SSB pairs since the RecG wedge domain and SSB IDL are unaltered in all of the two-hybrid pairs. To directly test RecG wedge domain residues that have been proposed to interact with SSB, plasmids encoding RecG F75A, M80A, R95A or F97D variants were substituted in the assay. Prior results from a co-purification assay suggested that these variants have severely reduced binding to SSB (49). In contrast with this earlier study, each of the strains grew on the selective medium, indicating that RecG wedge domain residues F75, M80, R95, and F97 are not required for interaction with SSB (Figure 3D).

**Figure 4.**
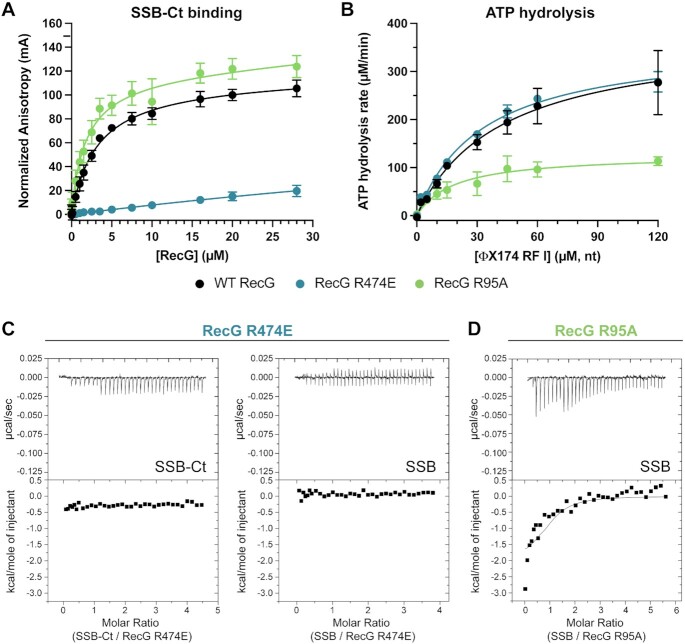
RecG R474E is functional as an ATPase but does not bind to SSB. (**A**) Fluorescence anisotropy binding isotherms for wildtype (black, data from Figure 2), R474E (blue), and R95A (green) RecG binding to a fluorescently labeled SSB-Ct peptide. (**B**) ATP hydrolysis rates for 150 nM wildtype (black), R474E (blue), or R95A (green) RecG with varying amounts of ΦΧ174 RF I DNA. The mean value of triplicate experiments is graphed with error bars representing one standard deviation from the mean. (**C**) ITC analysis of RecG R474E binding to the SSB-Ct (left) or full-length SSB (right). Thermograms (top) and binding isotherms (bottom) of titrations of SSB-Ct peptide, or full-length SSB into RecG R474E solutions. (**D**) ITC analysis of RecG R95A binding to SSB with thermogram (top) and binding isotherm (bottom) of titration of SSB into a solution of RecG R95A.

To more rigorously and quantitatively test the RecG HD2/SSB-Ct binding model, a RecG variant in which one of the putative SSB-binding residues is altered (RecG R474E) was purified and tested for binding to the SSB-Ct peptide and to full-length SSB *in vitro*. This variant was chosen because of the predicted interaction of the R474 side chain with the alpha-carboxyl group of SSB-Ct C-terminal Phe (Figure 3A). Analogous interactions have proven to be essential points of contact in other SIPs (10,19). The AlphaFold models and two-hybrid results predict that binding should be greatly reduced or eliminated by the mutation. Consistent with this prediction, the RecG R474E variant failed to bind the SSB-Ct peptide in the fluorescence anisotropy assay (Figure 4A). To rule out the possibility that the R474E sequence change caused misfolding of the protein that indirectly blocked SSB binding, DNA-dependent ATPase activity was measured for the RecG R474E variant and compared with wildtype RecG. Since the sequence change is in one of the two essential helicase domains of RecG, misfolding would result in significantly altered ATPase function. ATPase activity of RecG R474E was indistinguishable from that of wildtype RecG, with values for both maximal ATPase rates and the DNA concentrations required for 50% stimulation (*K*_DNA_) within error between the two proteins (Figure 4B and Table 2). Thus, we concluded that the RecG R474E variant was properly folded. To further examine the effect of the R474E sequence change, we also used ITC to measure RecG R474E binding to either the SSB-Ct or to full-length SSB. Binding was not detected in either titration (Figure 4C). These data confirm the predicted SSB binding site on the RecG HD2 domain.

**Table 2.** ATP hydrolysis kinetics parameters for wildtype and variant RecG proteins

RecG	*K*_DNA_ (μM nt)	*V*_max_ (μM/min)
WT	47.2 ± 8.3	379 ± 33
R474E	39.9 ± 2.9	363 ± 13
R95A	18.5 ± 4.9	127 ± 11

To directly test whether the proposed RecG wedge domain IDL binding site is important for interaction with SSB, we purified a RecG R95A variant and measured its activity *in vitro*. The R95A variant was selected because it showed the greatest (24-fold) reduction in co-purification with SSB in a previous study (49). We first measured binding to the SSB-Ct. The RecG R95A variant bound the SSB-Ct peptide in the fluorescence anisotropy assay with a *K*_D_ of 1.8 ± 0.4 μM, which is slightly tighter than wildtype RecG (3.1 ± 0.4 μM) (Figure 4A and Table 1). RecG R95A binding to the SSB-Ct was expected since our experiments had shown that the peptide bound at a site in the RecG HD2 domain. Interestingly, the DNA-dependent ATP hydrolysis activity of the RecG R95A variant differed from wildtype RecG, with a maximal ATPase rate of 127 ± 11 μM ATP/min, compared to 379 ± 33 μM ATP/min for wildtype RecG (Figure 4B and Table 1). This difference could be due to compromised DNA binding in the R95A variant. Arg95 is located on the DNA binding surface of the RecG wedge domain (74), and changing adjacent residues Phe96 or Phe97 to Ala results in drastic reductions in DNA binding and unwinding activity (91).

Although the RecG R95A wedge domain variant retained SSB-Ct binding and was able to interact with SSB in a two-hybrid assay, it was possible that the wedge domain plays a role in binding to the IDL in full-length SSB. To test this possibility, we used ITC to measure RecG R95A binding to full-length SSB (Figure 4C and Table 1). In contrast to a possible role for the wedge domain in SSB binding, titration of SSB with RecG R95A produced a remarkably similar binding isotherm to that of wildtype RecG. The data were fit to a one-site model with a fixed interaction stoichiometry of 1.0, revealing a ΔG° for RecG R95A binding to full-length SSB (−7.9 ± 0.2 kcal/mol) that was indistinguishable from that of wildtype RecG (−7.9 ± 0.3 kcal/mol). These data are not consistent with a role for Arg95, or the proposed IDL binding site (49), in binding SSB.

### Interaction with SSB is critical for RecG function *in vivo*

To study the effect of disrupting the RecG/SSB interaction, isogenic *E. coli* MG1655 strains in which the *recG* gene was mutated to encode R474E, R467E, or R614E RecG variants were created using λ Red recombination (75). Control strains that deleted *recG* (Δ*recG*) or expressed the R484E RecG variant that retained interaction with SSB (*recG-R484E*) were also created. Western blot analysis of the strains showed that RecG levels were very similar in each except for the Δ*recG* strain where, as expected, no RecG protein was observed (Supplementary Figure S3). Thus, changes in cellular activity for the point mutants could not be due to differences in protein levels.

**Figure 5.**
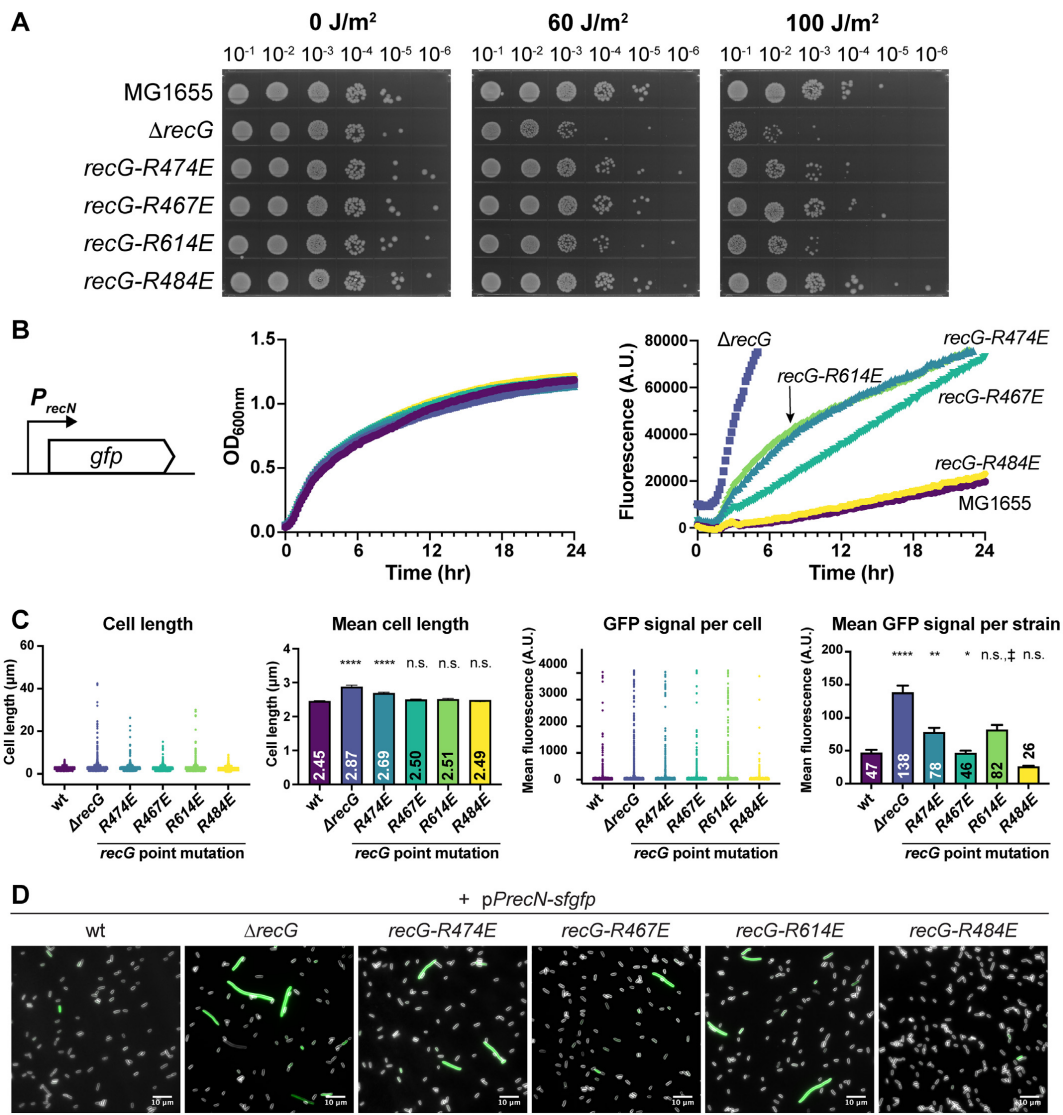
An interaction with SSB is important for RecG activity *in vivo*. (**A**) Spot plate assay showing growth of 10-fold serially diluted MG1655 (*recG*^+^), *ΔrecG*, or *recG* binding pocket mutant strains exposed to varying UV doses. (**B**) Liquid growth curves (center) and fluorescence signal (right) of MG1655 (*recG*^+^), *ΔrecG*, or *recG* binding pocket mutant strains carrying a plasmid with SuperGlo GFP under the control of the *recN* promoter (schematic, left). Experiments were completed in biological triplicate, with representative results shown from one replicate. (**C**) Cell length, mean cell length, GFP signal per cell, and mean GFP signal per strain measurements for MG1655 (*recG*^+^), *ΔrecG* or *recG* binding pocket mutant strains after 5 h of growth (stationary phase). At least 2000 cells were analyzed per strain. No significance (n.s.), *P*-value < 0.05 (*), *P*-value < 0.01 (**), *P*-value <0.0001 (****). ‡, although a much higher mean fluorescence was observed for the mutant over MG1655, the median fluorescence value and mean statistical test rank were similar, hence the result was not statistically significant. Bar graphs plot the average value across all cells measured for each strain with error bars representing the standard error of the mean. (D) Representative images of cells in the GFP channel for each *recG* binding pocket mutant and controls. The scale bar is 10 μM in length. Cell outlines from the FM4-64 channel are shown in white. Strains were imaged in biological triplicate on separate days.

We first examined whether the point mutant strains were sensitive to UV light, which is a DNA-damaging agent to which Δ*recG* cells are sensitive (72). Serial dilutions of the strains were plated on solid media, exposed to 0, 60 or 100 J/m^2^ UV light, and then grown to assess cell viability. In the absence of UV treatment, all of the strains plated with similar efficiencies, indicating that *recG* missense or deletion mutations were tolerated under normal growth conditions (Figure 5A). In contrast, treatment with 60 or 100 J/m^2^ UV light led to reduced plating efficiencies for the *recG-R474E*, *recG-R614E* and Δ*recG* strains. The *recG-R474E* and *recG-R614E* strains each displayed lower plating efficiencies than MG1655 (*recG^+^*), but they were not as sensitive as the Δ*recG* strain (Figure 5A). UV sensitivity of the *recG-R467E* strain was quite similar to MG1655. The *recG-R484E* control strain, which retains RecG/SSB binding, was not sensitized to UV light. Thus, UV sensitization was specific to cells harboring mutations that alter R474 or R614, both of which are involved in SSB-Ct binding. The result with the R467 variant suggests that this residue may play a more minor role in binding to SSB than R474 or R614 *in vivo*. Other SIPs have similarly displayed variation in the relative importance of each residue within the SSB-Ct binding site (10,19). These results indicate that the SSB-binding pocket in the HD2 domain is important for RecG DNA repair functions in *E. coli*.

When DNA damage accumulates in cells, the SOS response is initiated, resulting in upregulation of several DNA repair genes and error prone DNA polymerases (92,93). Strains lacking *recG* display constitutive induction of genes within the DNA-damage response SOS regulon in the absence of exogenous DNA damaging agents or conditions (72). To evaluate whether disruption of the RecG/SSB interaction also led to SOS induction, the *recG* mutant strains were transformed with a plasmid encoding SuperGlo GFP under the control of the *recN* promoter. *recN* expression is induced during the SOS response (94,95), making GFP expression from the plasmid an indicator of SOS induction. While all of the strains grew at similar rates, SOS induction was observed in the *recG-R467E*, *recG-R474E* and *recG-R614E* strains, with GFP levels well above those observed for MG1655 or *recG-R484E* control strains (Figure 5B). The levels were lower than those measured for the Δ*recG* strain, indicating that disruption of the RecG/SSB interaction does not eliminate all RecG functions in cells. As with UV sensitivity, SOS induction was less pronounced in the *recG-R467E* strain compared to the other *recG* mutant strains, further suggesting a more modest role for R467 in binding SSB than R474 or R614. These results further highlight the cellular importance of the RecG SSB-binding pocket for RecG function in *E. coli*.

The SOS response can lead to cell filamentation (96), which has been previously observed in Δ*recG E. coli* strains (73). To evaluate both cell length and individual cell SOS induction, microscope images of the transformed *recG* mutants in stationary phase (when differences in SOS induction are particularly pronounced) were acquired. The mean cell length measured for MG1655 cells was 2.45 ± 0.01 μm, which is similar to previously reported values (97). Filamentation was observed for each of the SSB-binding pocket mutant strains and the Δ*recG* strain, as evidenced by the presence of cells with lengths greater than 10 μm (Figure 5C, D). For *recG-R474E* and Δ*recG* cells, the mean cell length differed significantly from MG1655, whereas the number of non-filamented cells present in *recG-R467E* and *recG-R614E* strains reduced the significance level. Nonetheless, a larger number of filamented cells were observed for the *recG-R467E* and *recG-R614E* strains than in MG1655 or *recG-R484E* control strains (Figure 5C (left panel) and 5D). An increase in mean cell fluorescence was also observed for the Δ*recG*, *recG-R474E*, and *recG-R614E* strains. Interestingly, the majority of cells in each strain appear normal in morphology and are not induced for SOS, indicating that the absence of *recG* or loss of the RecG/SSB interaction does not lead to major defects for cells in every replication cycle.

### Cell expressing large chromosomally-encoded SSB IDL deletion variants are indistinguishable from wildtype *E*.*coli*

**Figure 6.**
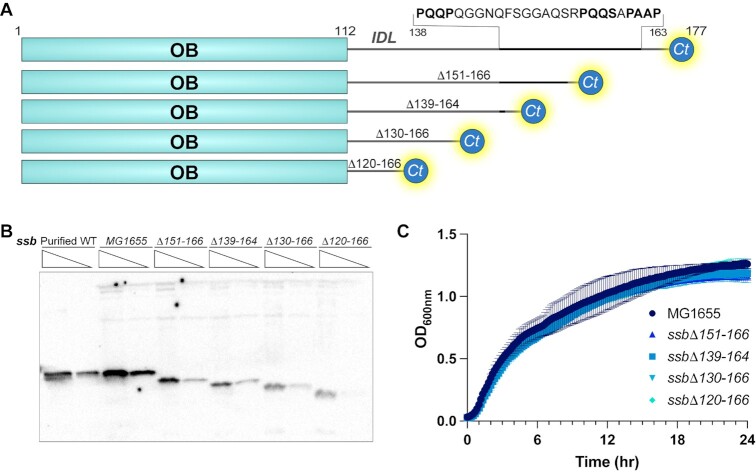
Cells expressing chromosomally encoded *ssb* IDL deletion mutants are viable. (**A**) Schematic diagram of SSB IDL deletion constructs. (**B**) Western blot probing for SSB in two dilutions of purified SSB and two dilutions of cell lysate samples from strains expressing wildtype *ssb*, *ssbΔ151–166*, *ssbΔ139–164*, *ssbΔ130–166*, or *ssbΔ120–166*. Upper bands present in cell lysate samples are due to antibody cross-reactivity. (**C**) Growth curves of MG1655 and *ssb* IDL deletion strains with the mean value for a set of experiments performed in biological triplicate graphed and error bars representing one standard deviation from the mean.

The simplest model that explains the results thus far is that RecG is a canonical SIP that binds directly to the SSB-Ct using a site in its HD2 domain and that neither the RecG wedge domain nor the SSB IDL are directly involved in RecG/SSB complex formation. These results led to a more general question – what is the cellular role, if any, for the SSB IDL in *E. coli*? To answer this question, a panel of isogenic *E. coli* MG1655 *ssb* mutant strains encoding SSB variants with large deletions in the IDL region was constructed (Figure 6A). These strains encoded SSB variants with deletions of residues 151–166 (*ssbΔ151–166*), 139–164 (*ssbΔ139–164*), 130–166 (*ssbΔ130–166*) or 120–166 (*ssbΔ120–166*). The *ssbΔ151–166* mutation removes two of the three PxxP motifs in the SSB IDL whereas the three remaining mutations remove all three PxxP motifs. Similar SSB variants have previously been shown to complement deletion of the essential *ssb* gene or a lethal *ssb* point mutation (41,42), however prior studies relied on SSB variants that were overexpressed on plasmids that could mask minor interaction defects with artificially elevated SSB protein levels.

We first used western blots to determine whether the different chromosomally encoded SSB IDL deletion variants supported viability in *E. coli* as the sole SSB in each strain. Possible *ssb* gene duplication events were a particular concern since prior attempts to fuse fluorescent domains into the C-terminus of *ssb* resulted in duplication events where a second *ssb* gene was added to the genome (98–101). Western blots of whole cell lysates confirmed that each strain encoded only the IDL deletion variant SSBs, demonstrating that removal of all SSB IDL PxxP motifs is tolerated in *E. coli* (Figure 6B). Whole-genome sequencing was also carried out for each strain to identify any suppressor mutations that could be needed to support viability in the *ssb* deletion mutants – no sequence differences were detected outside of the *ssb* IDL deletions.

Strains expressing the SSB IDL deletion variants produce weaker signals in the western blots (Figure 6B), which could be due to lower copy numbers for the variants and/or reduced reactivity with the SSB antibodies. Quantitative western blots were used to examine these possibilities by measuring the SSB levels in MG1655 and in the strain with the largest IDL deletion (*ssbΔ120–166*). Blots included purified protein standards to control for differences in antibody recognition between wildtype SSB and SSBΔ120–166. The analysis estimated that there were ∼19700 ± 5600 monomers of SSB per cell in MG1655 and ∼8800 ± 2700 monomers per cell in the *ssbΔ120–166* strain (Supplementary Figure S4). The estimates are similar to a previous report that measured ∼14400 SSB monomers per cell grown in rich media (102) and indicated that SSBΔ120–166 was present at a 2–3-fold lower copy number in the *ssbΔ120–166* strain than wildtype SSB in MG1655.

To examine whether deletion of the SSB IDL impacts DNA replication and cell growth rates, growth curves of the different *ssb* strains were measured and compared with MG1655 (*ssb*^+^). Each of the *ssb* mutant strains grew with kinetics that were indistinguishable from MG1655 (Figure 6C), indicating normal cellular function and DNA replication rates for each *ssb* mutant. This shows that large segments of the IDL can be deleted from *ssb* without impacting growth under normal culture conditions.

While all of the *ssb* mutant strains grew at similar rates, the strain carrying the largest deletion, *ssbΔ120–166*, exhibited slight SOS induction (Supplementary Figure S5), which was not observed for any of the other *ssb* IDL mutant strains. This result indicates that deletion of 47 of 57 IDL residues does produce a modest defect *in vivo*.

**Figure 7.**
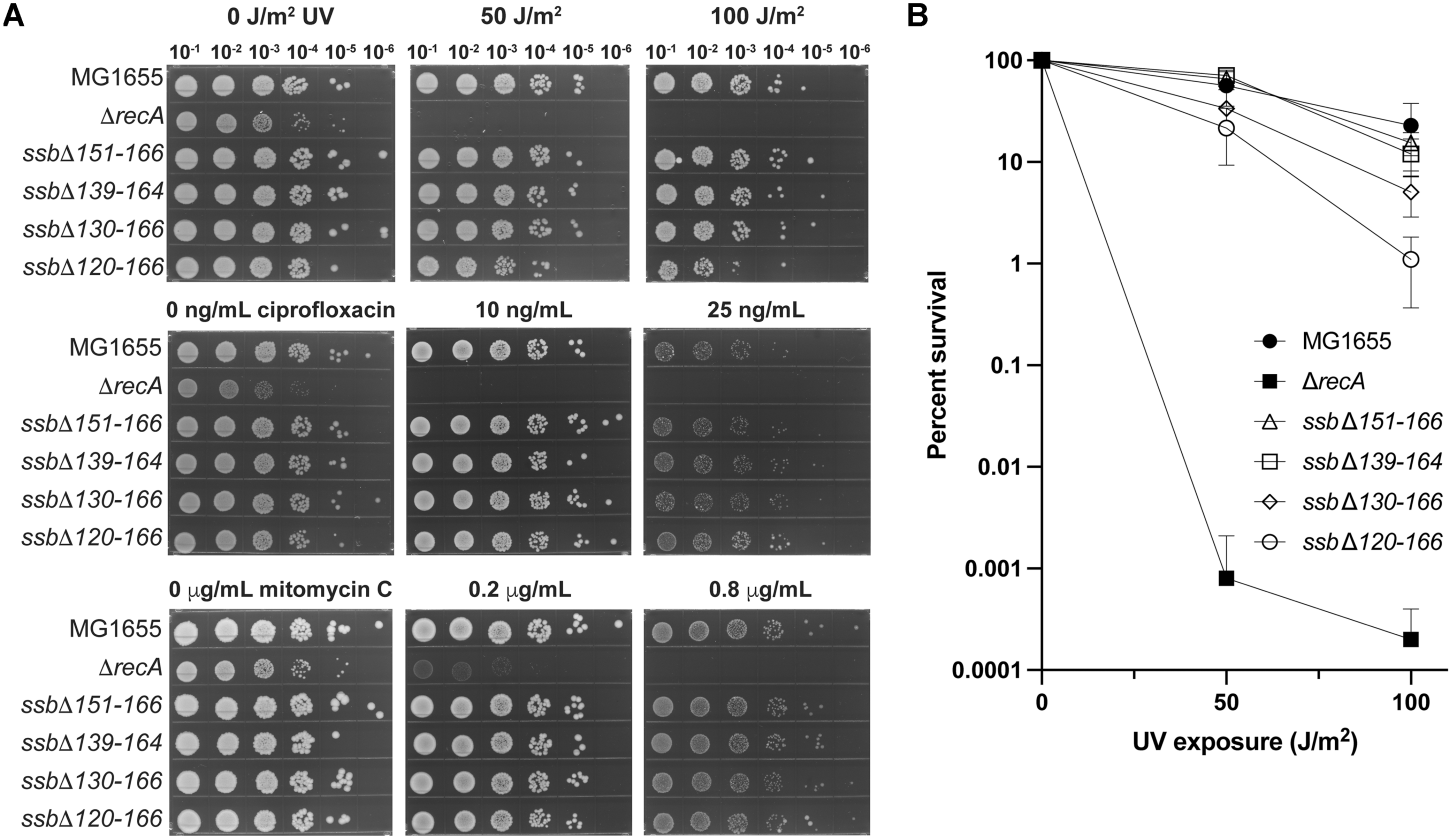
*ssb* IDL deletion mutant strains retain wildtype DNA repair capacity. (**A**) Spot plate assay showing growth of 10-fold serially diluted MG1655 (wildtype *ssb*), *ΔrecA*, *ssbΔ151–166*, *ssbΔ139–164*, *ssbΔ130–166*, and *ssbΔ120–166* strains exposed to varying UV (top), ciprofloxacin (center), or mitomycin C (bottom) doses. (**B**) Quantification of strain UV percent survival relative to CFU/ml of the strain at no UV exposure.

To test whether deletion of the SSB IDL affected DNA repair, the sensitivity of each of the *ssb* strains to UV light or to chemical DNA damaging agents (ciprofloxacin or mitomycin C) was examined and compared to MG1655 (Figure 7). A *ΔrecA* strain was included as a known DNA-damage sensitive control. For three of the *ssb* mutant strains (*ssbΔ151–166*, *ssbΔ139–164* and *ssbΔ130–166*), the cells displayed no change in DNA damage sensitivity compared to MG1655. Similar to the slight SOS induction we observed, the strain carrying the largest deletion, *ssbΔ120–166*, also displayed modest sensitization to UV light. This mutant was examined further in the next set of experiments. In total, these results show that *E. coli* grow and function normally with large IDL deletions, including deletions that remove all PxxP motifs from the SSB. These observations notably contrast with prior experiments showing that deletion of the SSB-Ct motif or C-terminal Phe residue is lethal in *E. coli* (41,42).

**Figure 8.**
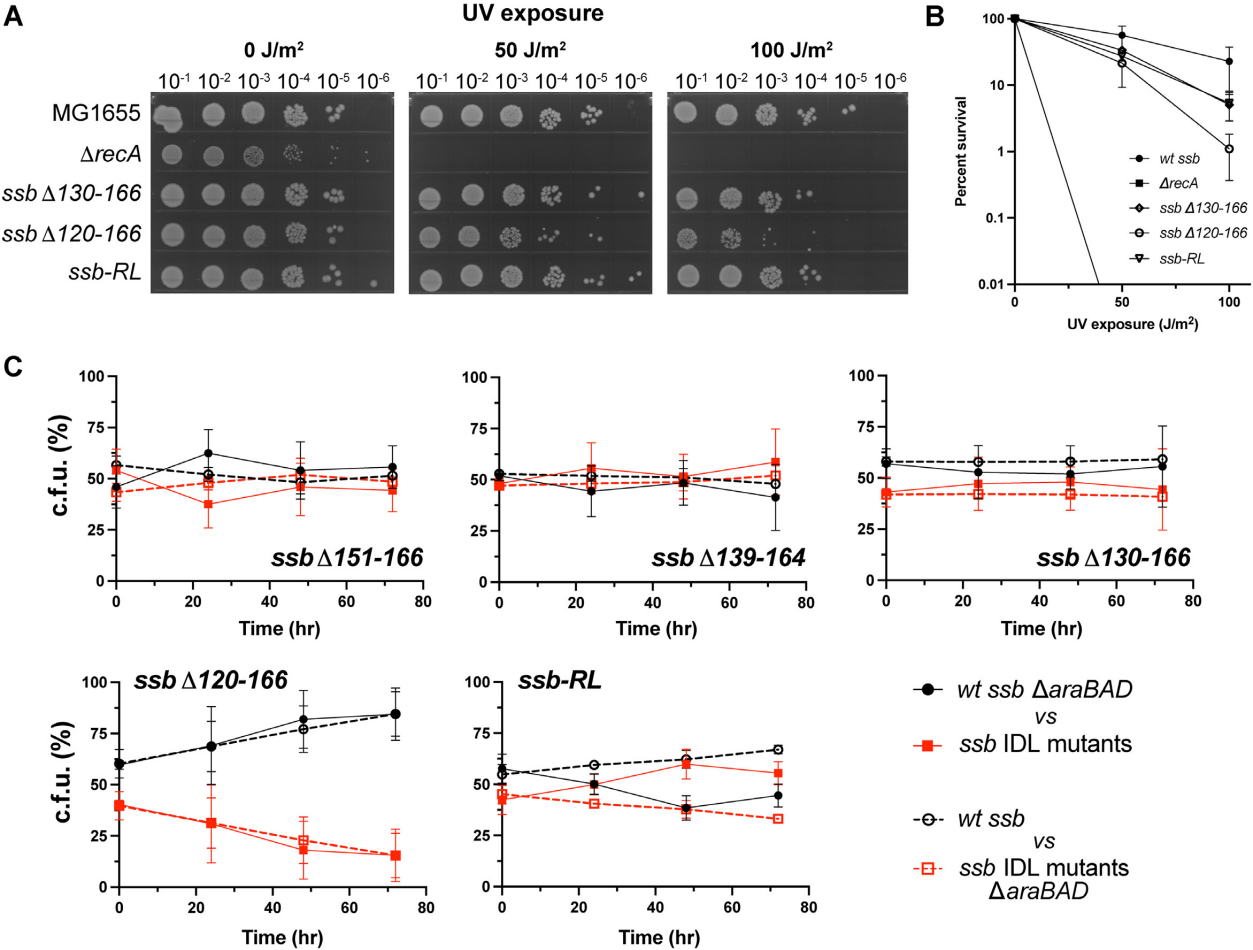
*E. coli* SSB requires a minimal IDL length, rather than a specific IDL sequence. (**A**) Spot plate assay showing growth of 10-fold serially diluted MG1655 (wildtype *ssb*), *ΔrecA*, *ssbΔ130–166*, *ssbΔ120–166*, or an *ssb-RL* strain exposed to varying UV. (**B**) Quantification of strain UV percent survival relative to CFU/ml of the strain at no UV exposure (data for *ssb-RL* added to survival graph from Figure 7B). (**C**) Percent of CFU of each strain from mixed cultures over three days of competitive growth. *ssb* IDL mutant strain data are plotted as red squares, and wildtype *ssb* results are shown as black circles. Competitions where the wildtype *ssb* strains are Δ*araBAD* are shown in solid connecting lines and filled markers. Competitions where the *ssb* IDL mutants are Δ*araBAD* are shown in dotted connecting lines and open markers. Average values of experiments completed in biological triplicate are plotted with error bars representing one standard deviation from the mean.

To further investigate the effect observed in the strain with the largest IDL deletion (*ssbΔ120–166*), we created a new mutant strain that added back residues 120–129 in a randomized sequence order (*ssb-RL*). We reasoned that if the specific sequence of residues 120–129 was important for SSB function, then the *ssb-RL* mutant should phenocopy the *ssbΔ120–166* strain. If instead residues 120–129 were important as a linker extending the distance between the OB domain and the SSB-Ct, then *ssb-RL* should phenocopy the *ssbΔ130–166* strain. Examination of the UV sensitivity and SOS response of the *ssb-RL* strain showed that it matched the sensitivity and SOS of the *ssbΔ130–166* strain, consistent with the simple spacer model for the IDL (Figure 8A and B, Supplementary Figure S5).

We further examined each of the *ssb* IDL mutants in competition growth assays, which are sensitive to very minor differences in activity for essential proteins. In this assay, two strains (*araBAD^+^* or Δ*araBAD*) are mixed in a single culture and the percent of cells from each starting strain is measured over time by counting the colonies that are white (*araBAD^+^*) or red (Δ*araBAD*). For each experiment, the *ssb* mutant or a complementary *ssb*^+^ strain was placed in either the *araBAD^+^* or Δ*araBAD* background and competitions were performed. The use of both versions of the competition controlled for any growth differences that might arise from the presence or absence of *araBAD*. For three of the *ssb* mutant strains (*ssbΔ151–166*, *ssbΔ139–164* and *ssbΔ130–166*), the cells displayed no fitness defects in competition assays against the matched *ssb*^+^ strain (Figure 8C). In contrast, the *ssbΔ120–166* strain was outcompeted by the *ssb*^+^ strain, consistent with its modest DNA damage sensitivity phenotype. The defect was complemented, however, in the *ssb-RL* strain. These data demonstrate that a minimum linker length of between 11 and 20 residues separating the SSB OB fold from the SSB-Ct is required for full SSB function, but the specific sequence of the linker is not important.

## DISCUSSION

This study leads to four major conclusions. First, the SSB-Ct is necessary and sufficient for interaction between SSB and the RecG helicase. No role was observed for SSB IDL PxxP motifs in binding to RecG. Second, a pocket on the RecG HD2 domain surface framed by Arg residues 467, 474 and 614 serves as the SSB binding site in RecG. Third, interaction between SSB and RecG is important for RecG function *in vivo*. Fourth, much of the SSB IDL can be deleted without affecting *E. coli* growth rates or repair of DNA damage caused by UV light, ciprofloxacin, or mitomycin C, although a minimal IDL length of 11–20 residues separating the OB domain and the SSB-Ct appears to be important *in vivo*.

SSB plays a critical role in cells by binding to ssDNA and interacting with ∼20 partner proteins involved in DNA replication, replication restart, repair, and recombination to coordinate their functions (1). With such a large network of SSB-protein interactions, the mechanisms by which SSB interacts with and differentiates among binding partners is a fundamental structural feature of bacterial genome maintenance. A large body of genetic, biochemical, biophysical, and structural evidence supports a shared mode of SSB recognition by SIPs where electrostatically similar binding pockets on each SIP interact with the highly conserved SSB-Ct (9,11–14,18–20,23,35,37,38,103,104). More recently, a second mode of SSB recognition has been proposed in which OB domains in RecG and in other SIPs interact with PxxP motifs within the SSB IDL (48,49,51, 54–57).

We found that *E. coli* RecG binds directly and specifically to the SSB-Ct, with no apparent contribution from the IDL. Studies from two complementary assays revealed 3–4 μM *K*_D_ values for the complex formed between RecG and the SSB-Ct or full-length SSB (Figure 2 and Table 1). Binding was sequence specific in both assays, with changes to the SSB-Ct sequence destabilizing or eliminating SSB interaction with RecG. Moreover, Δ*G*°, Δ*H*° and *T*Δ*S*° values observed for RecG binding to full-length SSB or to the SSB-Ct were nearly indistinguishable, strongly suggesting that the SSB-Ct comprises the complete binding site for RecG. In accordance with this finding, no binding was observed between RecG and a peptide that includes two of the PxxP motifs in SSB. These data are consistent with the SSB-Ct serving as the binding site for RecG as has been observed with other characterized SSB-protein interactions.

AlphaFold-Multimer was used to predict the structure of the RecG/SSB complex. The algorithm converged on an SSB-Ct interaction interface in the HD2 domain that was confirmed by both two-hybrid and biochemical assays (Figure 3). This site was distinct from a proposed PxxP IDL binding surface in the RecG wedge domain (48,49). Charge-reversal mutations to any of three Arg residues in the predicted binding pocket of RecG disrupted interaction with SSB, and, for a subset of the mutants, increased basal SOS response, and sensitized *E. coli* to UV light, indicating that the RecG interaction with SSB through a site in the RecG helicase domain plays an important role *in vivo* (Figures 3–5). In contrast, sequence changes in the RecG wedge domain did not disrupt binding to SSB.

The importance of the RecG/SSB interaction *in vivo* represents an intriguing new feature of RecG function. While the collective loss of SSB/SIP interactions in cells is lethal (41,42), the loss of any one SSB/SIP interaction may not produce strong phenotypes without additional enhancer mutations (104). Examples where loss of SSB interactions impact cellular functions of SIPs include the chi subunit of the DNA polymerase III holoenzyme, where a sequence change that destabilizes chi interaction with SSB leads to replisome instability and reduced cell growth rates, and DNA polymerase IV, where loss of interaction with SSB sensitizes cells to DNA damaging agents (19,30). As was observed for DNA polymerase IV, missense mutations in the *recG* SSB-interaction pocket produce measurable DNA repair defects, indicating that RecG activity is linked to its interaction with SSB. We note that not all charge reversal mutations had the same impact. Strains carrying charge-reversal changes to Arg474 and Arg614 in RecG appeared to confer the most significant phenotypic effects (Figure 5), which could indicate a greater importance for these residues in binding SSB. Such an arrangement aligns well with the proposed role for Arg474 in binding the SSB-Ct alpha-carboxyl group (Figure 3A), which has proven to be one of the most important points of contact for other SIPs (10,19), and for Arg614 in playing a primary role in binding to the SSB-Ct Asp side chains. We also note that the phenotypes of the binding pocket mutants are not as pronounced as the defects observed in Δ*recG* cells, indicating that some RecG functions are independent of interaction with SSB. Determining which functions are affected by the loss of the interaction is a question that remains to be answered. The cell length and SOS microscopy data reveal that only a sub-population of cells lacking *recG* entirely or expressing SSB binding pocket mutants suffer from deficiencies that lead to filamentation. This indicates that full RecG function is not normally needed during every cell cycle. However, when RecG does not function in situations where it is required, a deleterious situation occurs that is not readily reversed. Since RecG is estimated to be present at fewer than ten molecules per cell in *E. coli* (105), SSB-mediated localization to ssDNA substrates may be key for the specific functions of the RecG/SSB complex.

Due to the lack of an interaction between the SSB IDL and RecG, we extended our study to more broadly examine the importance of the IDL *in vivo* by generating chromosomally encoded *ssb* IDL deletion mutants. Unlike *ssb* mutants with deletions to the SSB-Ct motif, which are unable to replace *ssb* (41,42), several of the *ssb* IDL deletion mutants grew normally and were not significantly sensitized to DNA damaging agents (Figures 6 and 7). These included multiple strains that deleted all three of the PxxP motifs from the SSB IDL. The strain carrying the largest deletion to the IDL (*ssbΔ120–166*, which encodes an SSB variant with a 10-residue linker between the SSB OB domain and the SSB-Ct) appeared to be modestly sensitized to very high UV doses (100 J/m^2^) and displayed reduced fitness in competition growth assays. However, these defects were rescued when ten IDL residues in randomized sequence were added back into *ssb* (Figure 8). The simplest model that explains this result is that *E. coli* SSB requires a minimal length of 11–20 residues separating the SSB OB fold from the SSB-Ct for optimal function, and that the sequence of the spacer is not important. While the exact order of IDL residues does not appear to matter, previous work has demonstrated that IDL amino acid composition is important, as swapping the *E. coli* IDL sequence for the IDL sequence of *Plasmodium falciparum* SSB (an IDL with a higher ratio of charged residues) does not result in a fully functional SSB (50).

The *in vitro* characterization of the RecG/SSB interaction and the *in vivo ssb* mutant data provide strong evidence against a second mode of SSB recognition involving interactions with the SSB IDL. While it has been hypothesized that the IDL mediates a broad array of SSB/protein interactions, the RecG, RecO, and PriA proteins have specifically been proposed to participate in this binding mode (49). PriA structural (35) and biophysical (103,106) data show that its interaction with SSB is mediated by the SSB-Ct. Additionally, structural (20) and biophysical (106) data for RecO also support an interaction with the SSB-Ct. This latter investigation has revealed that RecO binds to the SSB-Ct and a second region of SSB, albeit not the IDL.

Previous investigations into the RecG-SSB interaction have primarily relied on experiments in which RecG and SSB variants simultaneously overexpressed in *E. coli* are co-purified using an affinity tag present on one of the two proteins (48,49). One issue that may confound interpretations from these experiments is that the concentrations of soluble RecG and SSB are not determined in the assay. Significant differences in expression of soluble RecG or SSB could alter co-purification without altering the stability of the RecG/SSB complex. In the experimental system used in prior reports (48,49), RecG and SSB are overexpressed from independent plasmids both of which rely on the pMB1 origin of replication, making them incompatible. Incompatibility can lead to variability in the copy number of each plasmid, thus altering protein abundance. Additionally, overexpression of SIPs can be toxic in *E. coli*, which may add selective pressure to reduce the concentrations of RecG variants in the overexpression system (30,107). Indeed, for DNA polymerase IV toxicity linked to its overexpression was used to identify a missense mutation within *dinB* that ablates its SSB-binding site (30). For these reasons, the present study has relied on purified proteins to identify the SSB-binding site on RecG.

The proposal that the SSB IDL mediates interactions with partner proteins postulates that SIPs exhibit an indirect dependence on the SSB-Ct for SSB binding because the conserved motif is important for the structural integrity of the IDL. However, structural data (31,36,108) and predictions (80) reveal that the IDL is unstructured, and the length of IDLs across bacterial species varies greatly (41). Indeed, prior studies have shown that the SSBΔ120–166 variant, lacking 47 of 57 IDL residues, retains the ability to bind SIPs (41). Similarly, SSB fluorescent fusion proteins where GFP or mTur2 have been introduced within the IDL retain SIP binding and are well tolerated in *E. coli* (53). We therefore conclude that the IDL does not play a direct role in SSB/SIP interactions.

If the IDL is not responsible for mediating SSB-protein interactions, does it have a role beyond acting as a bridge between the OB domain and SSB-Ct of SSB? Several possible roles have emerged from recent studies. *In vitro* investigations have uncovered a role for the IDL in regulating cooperative binding of SSB to DNA (41) and in mediating phase separation in SSB (50,52). Our results with IDL deletion mutants suggest that these activities may not be significant for normal cell growth and DNA repair, however there may be specialized situations that have not been examined in our study where such functions are important. Additionally, the role of the linker in tethering the OB fold to the SSB-Ct should not be overlooked. It is possible that the minimal linker length identified in our study is required in cells to prevent steric clashes between SIPs and the OB domain when SIPs are bound to the SSB-Ct. Also, the SSB-Ct is known to self-interact with the SSB OB domain (108), and the OB fold competes for SSB-Ct binding in variants with shorter linkers (106). The minimal IDL length of 11–20 residues may therefore be necessary to allow SSB/SIP interactions by mitigating SSB-Ct binding to the OB domain of SSB.

Our study has mapped the structural elements required for RecG/SSB complex formation and has demonstrated the importance of RecG/SSB interaction in *E. coli*. We have also shown that deletion of nearly all of the IDL region from SSB is well tolerated in *E. coli*. These studies lay the foundation for further studies examining specific situations in which RecG/SSB interaction are required and in determining whether the IDL could have roles beyond serving as a minimal length spacer in bacteria.

## DATA AVAILABILITY

Raw sequencing data for whole genome sequencing of strains EAW1515, EAW1516, EAW1517, EAW1518 and NJB48 can be found at NCBI SRA under BioProject ID PRJNA906490. The script used for microscopy image analysis and the setup file for cell outline recognition are provided.

## Supplementary Material

gkad162_Supplemental_Files
