# Supplementary material for: Interaction with the carboxy-terminal tip of SSB is critical for RecG function in E. coli
Source: Nucleic Acids Res. 2023 Mar 13;51(8):3735–53. doi: 10.1093/nar/gkad162 (PMC10164576; doi:10.1093/nar/gkad162)
Supplement: gkad162_Supplemental_Files [file gkad162_supplemental_files.zip › RecG SSB Supplementary Revised - clean.docx]

**SUPPLEMENTARY INFORMATION**

**Table S1.** Strains used in this study

| **Strain** | **Relevant genotype** | **Parent strain** | **Source/technique** |
| --- | --- | --- | --- |
| MG1655 | *wt ssb, wt recG*, *araBAD*^+^ | - | (1) |
| EAW20 | ∆*recA* | MG1655 | (2) |
| EAW214 | ∆*araBAD* | MG1655 | (2) |
| EAW505 | Δ*recG* | MG1655 | (3) |
| EAW1169 | *ssb-mTur2::kan* | MG1655 | (4) |
| EAW1515 | *ssb∆139-164::kan* | MG1655 | This study - λRED recombination |
| EAW1516 | *ssb∆151-166::kan* | MG1655 | This study - λRED recombination |
| EAW1517 | *ssb∆130-166::kan* | MG1655 | This study - λRED recombination |
| EAW1518 | *ssb∆120-166::kan* | MG1655 | This study - λRED recombination |
| EAW1650 | *recG-R474E::kan* | MG1655 | This study - λRED recombination |
| EAW1651 | *recG-R467E::kan* | MG1655 | This study - λRED recombination |
| EAW1693 | *recG-R614E::kan* | MG1655 | This study - λRED recombination |
| EAW1707 | *recG-R484E::kan* | MG1655 | This study - λRED recombination |
| NJB44 | ∆*araBAD* *ssb∆139-164::kan* | EAW214 | This study - P1 grown on EAW1515 |
| NJB45 | ∆*araBAD* *ssb∆151-166::kan* | EAW214 | This study - P1 grown on EAW1516 |
| NJB46 | ∆*araBAD* *ssb∆130-166::kan* | EAW214 | This study - P1 grown on EAW1517 |
| NJB47 | ∆*araBAD* *ssb∆120-166::kan* | EAW214 | This study - P1 grown on EAW1518 |
| NJB48 | *ssb-RL::kan* | MG1655 | This study - λRED recombination |
| NJB51 | Δ*araBAD* *ssb-RL::kan* | EAW214 | This study - P1 grown on NJB48 |

**Table S2**. Primers used in this study

| **Primer** | **Sequence (5' to 3')** | **Use** |
| --- | --- | --- |
| SSBafter | CATCCACCTTAAAACAATATAACCTATTGTTTT  AATGACAAAGTTAGCAG CCGGATCTCAGTGG | PCR for chromosomal incorporation |
| SSB d139-164 | CGGCAGGTGGCAATATCGGTGGTGGTCAGCCG  CAGGGCGGTTGGGGTCAGTCTAACGAGCCGCC  GATGGA | PCR for chromosomal incorporation |
| SSB d151-166 | GTTGGGGTCAGCCTCAGCAGCCGCAGGGTGGC  AATCAGTTCAGCGGCGGCCCGCCGATGGACTT  TGATGATG | PCR for chromosomal incorporation |
| SSB d130-166 | GTGGTCGTCAGGGTGGTGGCGCTCCGGCAGGT  GGCAATATCGGTGGTGGTCCGCCGATGGACTT  TGATGATG | PCR for chromosomal incorporation |
| SSB d120-166 | TGAACGTTGGCGGCACCATGCAGATGCTGGGT  GGTCGTCAGGGTGGTGGCCCGCCGATGGACTT  TGATGATGACA | PCR for chromosomal incorporation |
| SSB 120-129 rand | GTGGTGAACGTTGGCGGCACCATGCAGATGCT  GGGTGGTCGTCAGGGTGGTAACGGTGCTGGTG  CTCCGGGTGGTATCGGTGGCCCGCCGATGGAC  TTTG | PCR for chromosomal incorporation |
| recGafter | CCGGCAGGAAGGTAGGGTAACCTGAAATGGC  GGTCTTCTCACTGCCGCCTTGGCCACGATGCG  TCCGGCGTA | PCR for chromosomal incorporation |
| recGdsBam | GGATATCGGATCCTTACGCATTCGAGTAACGT  TC | PCR for chromosomal incorporation |
| recGend | CGAAGCTGATGCCGACTGGTGGGCTACTATG  CAGGCTGCAGGGTAAGTGCCATGAAAGGTCG  CCTGTTAGATG | PCR for chromosomal incorporation |
| recGus | GAACGCTGGATGCCGGAGACGGAACGTTACT  CGAATGCGTAACCCTTTCGT CTTCAAGAATTC | PCR for chromosomal incorporation |
| recG R474 rev2 | TTCGTCAATGATGTCGGTACGGCG | IVA mutagenesis |
| recG R474E for | GTACCGACATCATTGACGAAGTGCACCACGC  CTGCATAAC | IVA mutagenesis |
| recG R467E for | GTCGCTATTCCTGATACCGAACGTACCGACA  TCATTGACCGC | IVA mutagenesis |
| recG R467 rev | GGTATCAGGAATAGCGACCGTAGTCA | IVA mutagenesis |
| recG R484E for | CTGCATAACTGAAGGTGAACAGGCATACTG  GGTTTGTACGTTG | IVA mutagenesis |
| recG R484 rev | ACCTTCAGTTATGCAGGCGTG | IVA mutagenesis |
| recG R614E for | CCTGCAAGTGCTGGAAGACAGTAACGACGG  TTTTGTGATTGC | IVA mutagenesis |
| recG R614 rev | CAGCACTTGCAGGCGAATTTG | IVA mutagenesis |

**Table S3.** Plasmids used in this study

| **Plasmid** | **Description** | **Use** |
| --- | --- | --- |
| pQBI63 | SuperGlo GFP under the control of the T7 promoter | SOS induction control plasmid |
| pGAD-C3 | empty pGAD vector | Y2H negative control |
| pGBD-C3 | empty pGBD vector | Y2H negative control |
| pT7pol26 | Expression of T7 RNA polymerase for T7 promoter-driven protein expression | Protein expression |
| pEAW111 | *recG* in pET21d | WT RecG expression |
| pEAW134 | *ssb* in pET21a | WT SSB expression |
| pEAW903 | SuperGlo GFP under the control of the *recN* promoter | SOS induction plasmid |
| pEAW1156 | *recG* in pGBD (Gal4 DB fused to RecG N-terminus) | Y2H |
| pEAW1158 | *recG* in pGAD (Gal4 AD fused to RecG N-terminus) | Y2H |
| pEAW1292 | *recG R474E* in pGBD, made by IVA mutagenesis of pEAW1156 | Y2H |
| pEAW1293 | *recG R467E* in pGBD, made by IVA mutagenesis of pEAW1156 | Y2H |
| pEAW1294 | *recG R484E* in pGBD, made by IVA mutagenesis of pEAW1156 | Y2H |
| pEAW1320 | *recG F75A* in pGBD, made by IVA mutagenesis of pEAW1156 | Y2H |
| pEAW1321 | *recG M80A* in pGBD, made by IVA mutagenesis of pEAW1156 | Y2H |
| pEAW1322 | *recG R95A* in pGBD, made by IVA mutagenesis of pEAW1156 | Y2H |
| pEAW1323 | *recG F97D* in pGBD, made by IVA mutagenesis of pEAW1156 | Y2H |
| pEAW1324 | *recG R95A* in pET21d, made by IVA mutagenesis of pEAW111 | RecG R95A overexpression |
| pSW044 | *ssb* in pGAD (Gal4 AD fused to SSB N-terminus) | Y2H |
| pSW045 | *ssb* in pGBD (Gal4 BD fused to SSB N-terminus) | Y2H |
| pNJB1 | *recG R614E* in pGBD, made by IVA mutagenesis of pEAW1156 | Y2H |
| pNJB3 | *recG R474E* in pET21d, made by IVA mutagenesis of pEAW111 | RecG R474E expression |


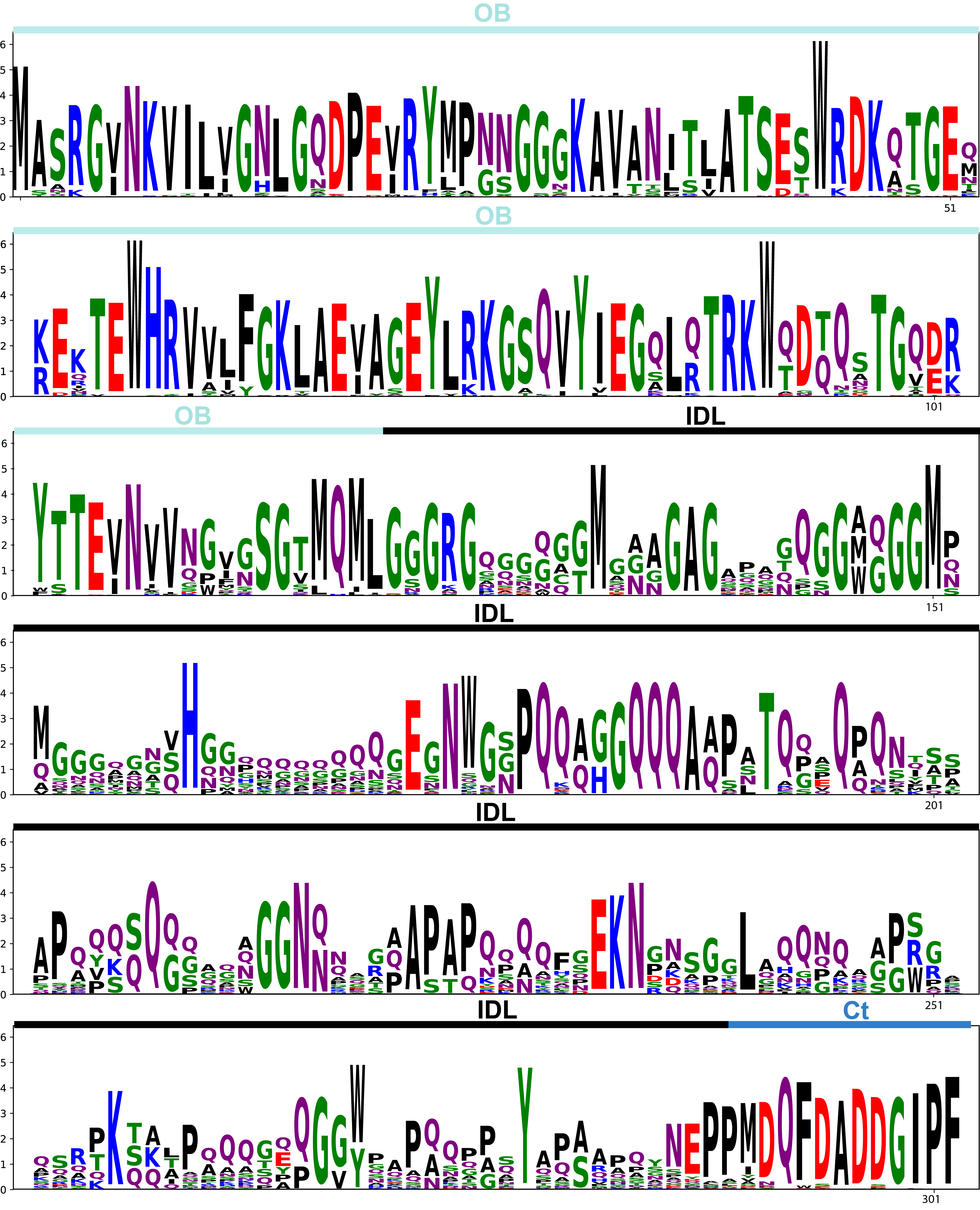


**Figure S1. Bacterial SSB conservation analysis.** Conservation data (OB = light cyan, IDL = black, SSB-Ct = marine) obtained with ConSurf (5,6) of 300 closest homologs to *E. coli* SSB. Sequence logo generated with Logomaker (7).

**
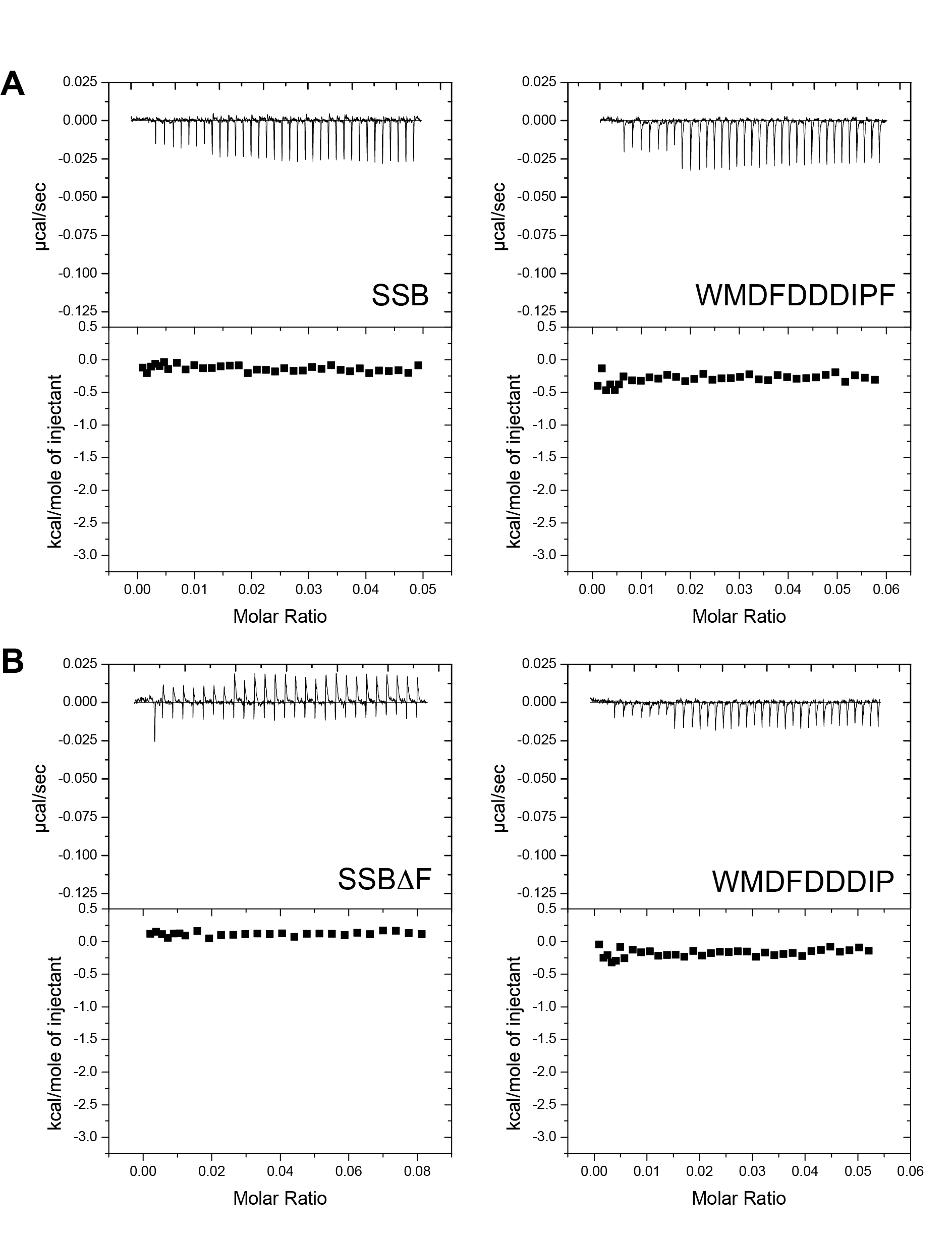
**

**Figure S2. ITC heat of dilution thermograms and binding isotherms.** (A) ITC data for titrations of full-length SSB (left) and SSB-Ct peptide (right) into ITC buffer. (B) ITC data for titrations of full-length SSBΔF (left) and ΔF peptide (right) into ITC buffer.


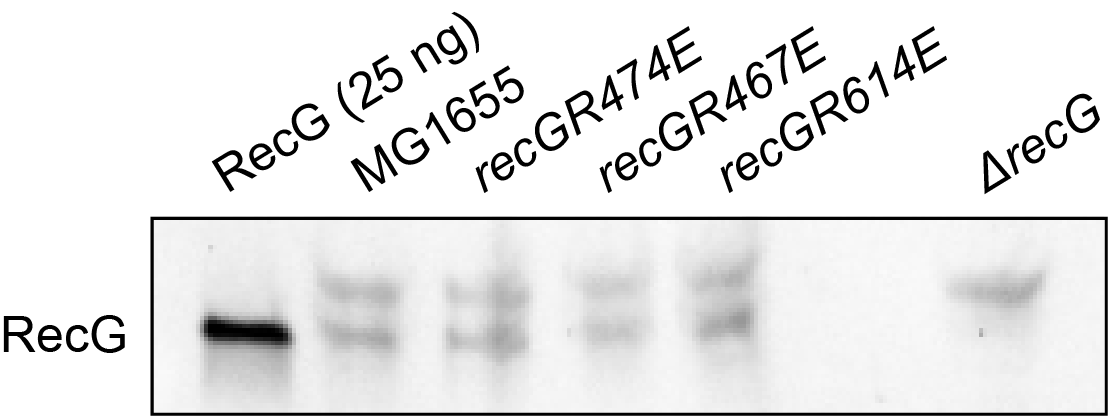


**Figure S3.** **Expression of *recG* mutants.** RecG western blot of purified protein and cell lysate samples from strains expressing WT/variant RecG or lacking RecG. The top band present in cell lysate samples is due to antibody cross-reactivity.

**
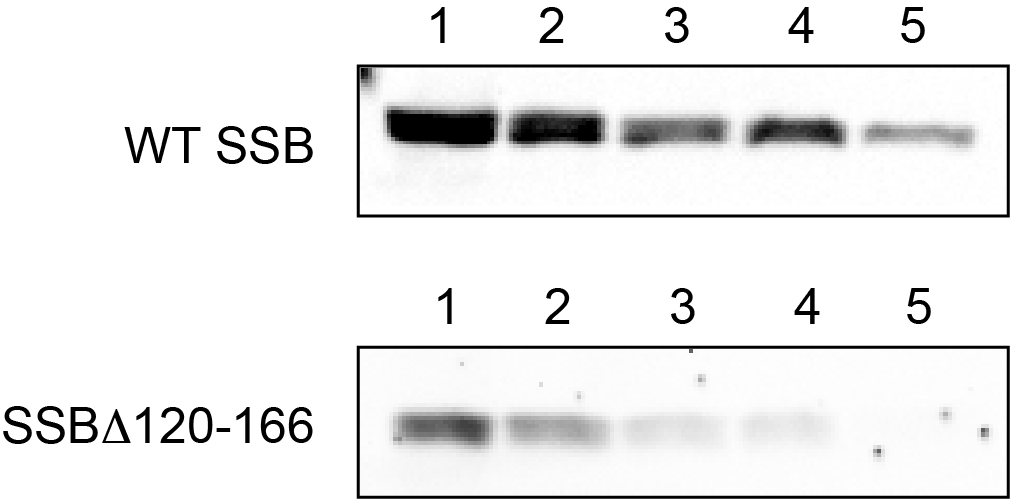
**

**Figure S4. Quantification of SSB by western blot.** Lanes 1-3 contain 10 μL of 0.1, 0.05, and 0.025 μM purified protein (WT SSB or SSBΔ120-166) standards, respectively. Lane 4 contains 10 μL of undiluted cell lysate samples from MG1655 (expresses WT SSB) or EAW1518 (expresses SSBΔ120-166). Samples loaded in lane 4 were diluted 1:3 and 10 μL were loaded in lane 5. Bands in lane 4 were used to quantity SSB expression by comparison of band intensity to signal from purified standard samples.

**
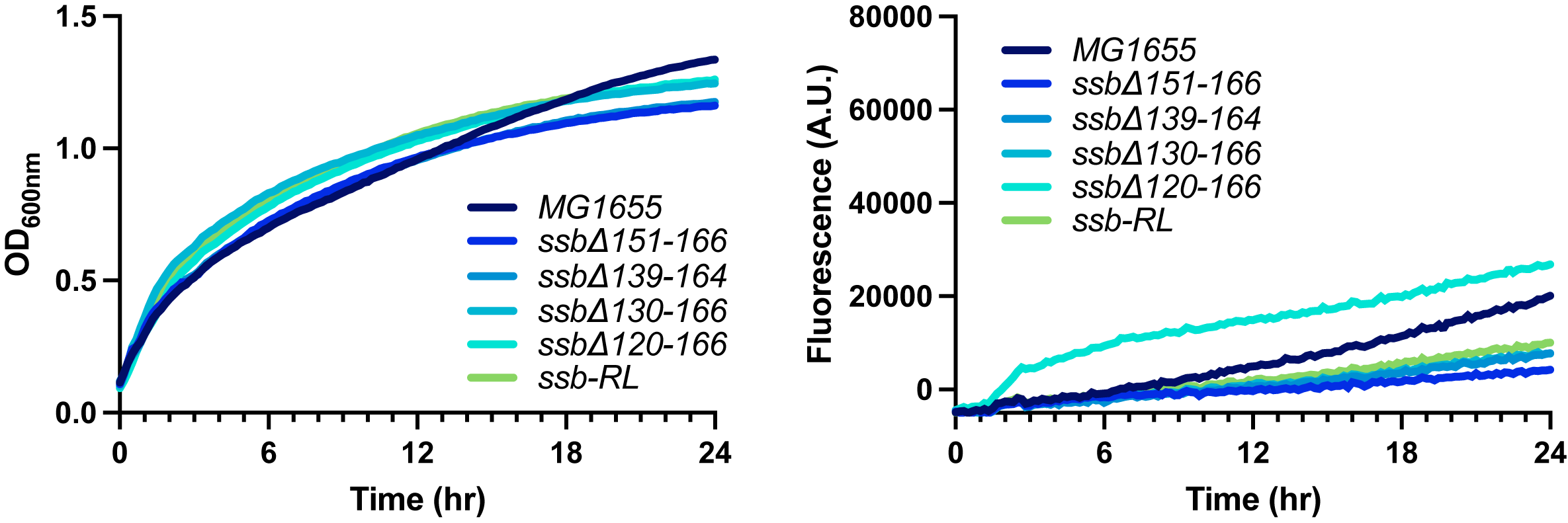
**

**Figure S5. Comparison of *ssb* IDL mutant strain growth rate kinetics and SOS induction.** Liquid growth curves (left) and fluorescence signal (right) of MG1655 (*ssb*^+^) or *ssb* IDL mutant strains carrying a plasmid with SuperGlo GFP under the control of the *recN* promoter. Experiments were completed in biological triplicate, with representative results shown from one replicate. Strong SOS induction signal can be observed in Figure 5B for comparison.

**Supplementary Information References**

1. Blattner, F.R., Plunkett, G., Bloch, C.A., Perna, N.T., Burland, V., Riley, M., Collado-Vides, J., Glasner, J.D., Rode, C.K., Mayhew, G.F. *et al.* (1997) The Complete Genome Sequence of Escherichia coli K-12. *Science*, **277**, 1453-1462.

2. Kim, T., Chitteni-Pattu, S., Cox, B.L., Wood, E.A., Sandler, S.J. and Cox, M.M. (2015) Directed Evolution of RecA Variants with Enhanced Capacity for Conjugational Recombination. *PLOS Genetics*, **11**, e1005278.

3. Romero, Z.J., Chen, S.H., Armstrong, T., Wood, E.A., van Oijen, A., Robinson, A. and Cox, M.M. (2020) Resolving Toxic DNA repair intermediates in every E. coli replication cycle: critical roles for RecG, Uup and RadD. *Nucleic Acids Research*, **48**, 8445-8460.

4. Dubiel, K., Henry, C., Spenkelink, L.M., Kozlov, A.G., Wood, E.A., Jergic, S., Dixon, N.E., van Oijen, A.M., Cox, M.M., Lohman, T.M. *et al.* (2020) Development of a single-stranded DNA-binding protein fluorescent fusion toolbox. *Nucleic Acids Research*, **48**, 6053-6067.

5. Ashkenazy, H., Abadi, S., Martz, E., Chay, O., Mayrose, I., Pupko, T. and Ben-Tal, N. (2016) ConSurf 2016: an improved methodology to estimate and visualize evolutionary conservation in macromolecules. *Nucleic Acids Research*, **44**, W344-W350.

6. Glaser, F., Pupko, T., Paz, I., Bell, R.E., Bechor-Shental, D., Martz, E. and Ben-Tal, N. (2003) ConSurf: Identification of Functional Regions in Proteins by Surface-Mapping of Phylogenetic Information. *Bioinformatics*, **19**, 163-164.

7. Tareen, A. and Kinney, J.B. (2019) Logomaker: beautiful sequence logos in Python. *Bioinformatics*, **36**, 2272-2274.
